# Dietary fibre-adapted gut microbiome clears dietary fructose and reverses hepatic steatosis

**DOI:** 10.1038/s42255-025-01356-0

**Published:** 2025-09-15

**Authors:** Sunhee Jung, Hosung Bae, Won-Suk Song, Yujin Chun, Johnny Le, Yasmine Alam, Amandine Verlande, Sung Kook Chun, Joohwan Kim, Miranda E. Kelly, Miranda L. Lopez, Sang Hee Park, Daniel Onofre, Jongwon Baek, Ki-Hong Jang, Varvara I. Rubtsova, Alexis Anica, Selma Masri, Gina Lee, Cholsoon Jang

**Affiliations:** 1 Department of Biological Chemistry, School of Medicine, University of California Irvine, Irvine, CA USA; 2 Department of Microbiology and Molecular Genetics, School of Medicine, University of California Irvine, Irvine, CA USA; 3 Chao Family Comprehensive Cancer Center, University of California Irvine, Irvine, CA USA; 4 Center for Epigenetics and Metabolism, University of California Irvine, Irvine, CA USA

**Keywords:** Small intestine, Metabolomics, Metabolism, Liver, Microbiota

## Abstract

Excessive consumption of the simple sugar fructose, which induces excessive hepatic lipogenesis and gut dysbiosis, is a risk factor for cardiometabolic diseases. Here we show in male mice that the gut microbiome, when adapted to dietary fibre inulin, catabolizes dietary fructose and mitigates or reverses insulin resistance, hepatic steatosis and fibrosis. Specifically, inulin supplementation, without affecting the host’s small intestinal fructose catabolism, promotes the small intestinal microbiome to break down incoming fructose, thereby decreasing hepatic lipogenesis and fructose spillover to the colonic microbiome. Inulin also activates hepatic de novo serine synthesis and cystine uptake, augmenting glutathione production and protecting the liver from fructose-induced lipid peroxidation. These multi-modal effects of inulin are transmittable by the gut microbiome, where *Bacteroides acidifaciens* acts as a key player. Thus, the gut microbiome, adapted to use inulin (a fructose polymer), efficiently catabolizes dietary monomeric fructose, thereby protecting the host. These findings provide a mechanism for how fibre can facilitate the gut microbiome to mitigate the host’s exposure to harmful nutrients and disease progression.

## Abstract (web summary)

The dietary fibre inulin is shown to promote fructose catabolism by the small intestinal microbiome, thereby mitigating fructose-induced hepatic lipogenesis and steatosis.

## Main

Driven by the increased consumption of simple sugar and fat, the prevalence of metabolic diseases, including obesity, diabetes and metabolic dysfunction-associated steatotic liver disease (MASLD), has risen alarmingly over the past few decades^1^. High-fat-containing diets have been extensively used in animal models to study the mechanism of obesity-associated MASLD^2,3^. Importantly, ~25% of patients with MASLD do not have obesity, but they show even higher risks than patients with obesity of developing severe metabolic dysfunction-associated steatohepatitis (MASH), cirrhosis and hepatocellular carcinoma^4^ because they miss timely screening owing to their normal body weights. Moreover, although patients with MASLD exhibit a twofold higher risk of developing almost all types of cancers, this risk is not observed in patients with MASLD who are not obese^5,6^, further underscoring the importance of studying MASLD irrespective of obesity.

High fructose corn syrup (HFCS) consumption, especially in liquid form (for example, soft drinks, juice), is an established risk factor for lean MASLD, MASH, cirrhosis and hepatocellular carcinoma^7–10^. Prior studies with various nutritional interventions and genetic animal models revealed many harmful effects of fructose on bodily health, mainly through metabolic alterations in the liver, gastrointestinal system and colonic gut microbiota, where most fructose catabolism occurs^11–16^. This toxic feature of fructose is associated with its unique metabolism in mammals^17^. Unlike glycolysis, which possesses several rate-limiting enzymes and product feedback inhibitions, fructolysis is mediated by ketohexokinase, which is not allosterically regulated, and triokinase^18,19^. Therefore, when tissues that express ketohexokinase encounter fructose, they rapidly phosphorylate fructose to fructose 1-phosphate and deplete intracellular ATP. Moreover, cleavage of fructose 1-phosphate generates glyceraldehyde, a reactive metabolite that can induce oxidative stress and damage DNA, RNA and proteins^20,21^. In addition, compared to glucose, fructose is a much more potent transcriptional activator of lipogenesis in the liver, inducing hepatic steatosis and insulin resistance^22–24^. Finally, excessive fructose intake is linked to intestinal abnormalities, including extended intestinal villi, impaired barrier functions and gut dysbiosis^25–27^.

**Fig. 1:**
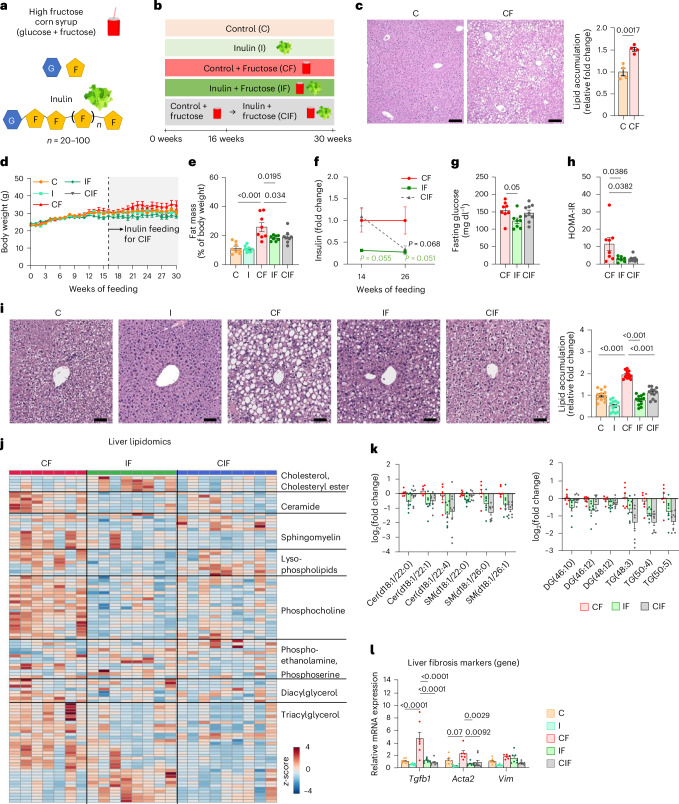
Reversal of HFCS-induced metabolic dysfunctions by inulin supplementation. **a**, Chemical structures of HFCS and inulin. G, glucose; F, fructose. **b**, Experimental groups. Mice received a control (C) or inulin-supplemented (I) diet with or without HFCS (F) in drinking water. For the CIF group, mice first received a control diet with HFCS and then an inulin diet with HFCS from week 16. **c**, Representative liver H&E staining and quantitation of lipid accumulation. Scale bars, 200 μm (*n* = 4, 4 mice). **d**,**e**, Body weight (**d**) and fat mass (**e**) (*n* = 8, 8, 8, 8, 9 mice). **f**–**h**, Fasting insulin on weeks 14 and 26 (**f**) (*n* = 8, 7, 8 mice), fasting glucose on week 26 (**g**) (*n* = 8, 8, 9 mice) and HOMA-IR on week 26 (**h**) (*n* = 7, 7, 8 mice). **i**, Representative liver H&E staining and quantitation of lipid accumulation. Staining was performed in three mice per group, and lipid accumulation was quantified in four randomly selected areas per liver. Scale bars, 50 μm. **j**, Liver lipidomics (*n* = 7, 8, 9 mice). **k**, Abundances of the indicated hepatic lipid species normalized to the CF group (*n* = 7, 8, 9 mice). Cer, ceramide; SM, sphingomyelin; DG, diacylglycerol; TG, triacylglycerol. Numbers in brackets denote total number of carbon atoms and number of double bonds. **l**, Liver fibrosis marker gene expression (*n* = 8, 8, 7, 7, 9 mice). Data are means; error bars, s.e.m. *P* values determined by one-way ANOVA with Tukey’s honestly significant difference (HSD) test (**e**–**l**) or two-sided unpaired Student’s *t*-test (**c**). Illustrations in **a** and **b** created with BioRender.com. Source data

In contrast to the monomeric sugar fructose, inulin, a fructose polymer (see Fig. 1a), has been used as a prebiotic fibre that can improve insulin sensitivity in patients with diabetes and reduce cholesterol and triglyceride levels in individuals with obesity^28–30^. Consistently, mice fed inulin with a MASH-inducing diet exhibit less severe steatohepatitis phenotypes than mice fed the MASH diet alone^31^. Given that the gut microbiome, primarily in the large intestine, is responsible for digesting inulin, many studies have focused on changes in colonic microbiome composition induced by inulin^31,32^, which increases short-chain fatty acid (SCFA) production and has beneficial effects^33^. However, SCFAs are also copiously produced from many other nutrients, including proteins and fructose^15,34^, and excessive SCFAs can contribute to hepatic lipogenesis and worsen metabolic disorders through the gut–brain axis^15,35^. Thus, the protective effects of inulin probably involve further mechanisms in addition to colonic microbial changes and SCFA production.

Using in vivo isotope tracing, metabolomics and transcriptomics analysis in mice, we report that inulin induces the small intestinal microbiome to clear dietary fructose, thereby reducing the detrimental effects of fructose on the host. In addition, inulin redirects fructose-derived carbons toward de novo serine and glutathione synthesis in the liver to suppress lipid peroxidation induced by fructose. Finally, through microbiome sequencing, antibiotic treatment, gut microbiota transplantation and bacterial inoculation experiments, we verified the essential role of the gut microbiome in these multiple effects of inulin and identified *Bacteroides* *acidifaciens* as a key mediator. Our findings thus provide a previously unrecognized mechanism by which the fibre-modulated gut microbiome can eliminate deleterious dietary nutrients and protect the host.

## Results

### Reversal of fructose-induced metabolic dysfunctions by inulin supplementation

To recapitulate the phenotype of lean patients with MASLD, we fed mice HFCS-containing drinking water with standard chow. In parallel, to determine the effect of inulin supplementation on HFCS-elicited pathologies, we used an open-source diet, a control diet used in many nutritional studies^36–38^, and formulated an inulin-enriched diet by replacing a small portion of corn starch (10% w/w) with inulin, as in previous studies^39–41^ (Supplementary Table 1). This amount of inulin is higher than the ~4% w/w that is typically tolerated by humans^42^, whereas rodent studies use 10% or even higher (15%)^43–46^, given that the metabolic rate and food consumption are higher in rodents than humans^47^. We also sought to test whether delayed inulin supplementation can reverse already established MASLD (CIF group in Fig. 1b). To achieve this goal, we first fed mice a control diet with HFCS-water for 16 weeks to induce hepatic steatosis (Fig. 1c). We then switched the diet to the inulin-supplemented diet with continual HFCS provision for an additional 14 weeks.

**Extended Data Fig. 1:**
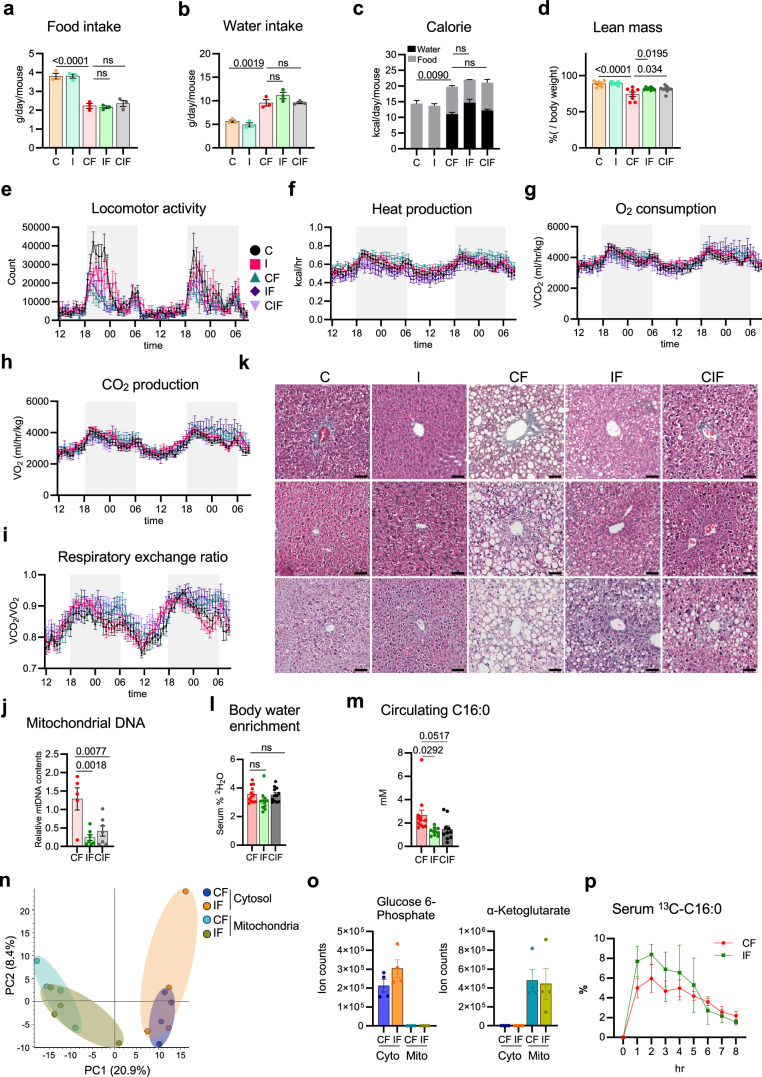
Metabolic characterization of mice fed HFCS, inulin or combinations. **a-c**, Daily food, water, and calorie intake (n = 3,3,3,3,3 cages). **d**, Lean mass normalized to body weight (n = 8,9,8,8,9 mice). **e-i**, Locomotor activity, heat production, O_2_ consumption, CO_2_ production, and respiratory exchange ratio (n = 8,9,8,8,9 mice). **j**, Hepatic mtDNA contents (n = 5,7,7 mice). **k**, Representative liver trichrome staining for fibrosis measurements from two independent experiments. Scale bars, 50 μm (n = 3,3,3,3,3 mice). **l**, body water enrichment measured 15 hours after administration of ^2^H_2_O. **m**, Circulating total saponified palmitate concentration (n = 12,9,10 mice). **n**, PCA plot of metabolites in cytosol and mitochondria fractions of liver (n = 4,4 mice). **o**, Glucose-6-phosphate and α-ketoglutarate levels in cytosolic vs mitochondrial fractions of liver (n = 4,4 mice). **p**, ^13^C-labeled circulating palmitate fraction over time after oral provision of ^13^C-palmitate (n = 4,3 mice). Data are mean±s.e.m. P-values by one-way ANOVA with Tukey’s HSD test. Source data

Substituting corn starch for inulin resulted in ~7% fewer calories (Supplementary Table 1). However, calculation of total calorie intake based on the consumption of chow and HFCS (Extended Data Fig. 1a,b) indicated no significant difference in total calorie intake between the three groups that received HFCS (Extended Data Fig. 1c). Consistent with previous reports^16,48^, without a high-fat diet provision, HFCS drinking alone did not increase body weight substantially (Fig. 1d), although it increased the per cent fat mass (Fig. 1e) and decreased per cent lean mass (Extended Data Fig. 1d) compared to the control diet alone. Intriguingly, both simultaneous and delayed inulin supplementation suppressed the HFCS-induced fat–lean mass imbalance (Fig. 1e and Extended Data Fig. 1d). However, this effect of inulin was not a result of changes in locomotive activity, heat production, metabolic rates or respiratory exchange ratio (Extended Data Fig. 1e–i).

We next examined the effect of simultaneous and delayed inulin supplementation on insulin resistance and hepatic steatosis elicited by HFCS drinking. Compared to HFCS feeding alone, HFCS and inulin co-feeding showed decreased trends in fasting insulin and glucose levels (Fig. 1f,g). Switching the control diet to the inulin-enriched diet (the CIF group), despite continued HFCS drinking, also tended to decrease fasting insulin levels (Fig. 1f) without affecting fasting glucose levels (Fig. 1g). Driven by the decreased insulin levels, delayed inulin supplementation reduced the homoeostatic model assessment of insulin resistance (HOMA-IR) as much as simultaneous inulin supplementation did (Fig. 1h).

Next, we examined the effect of inulin on the liver. Simultaneous and delayed inulin supplementation suppressed or reversed HFCS-induced hepatic lipid accumulation (Fig. 1i). Liver lipidomics analysis also revealed that inulin provision reduced hepatic lipid species, including ceramide, sphingomyelin, diacylglycerol and triacylglycerol (Fig. 1j,k). In patients with MASLD, mitochondrial DNA levels are generally increased as a compensatory mechanism in response to mitochondrial damage^49,50^. Inulin supplementation decreased mitochondrial DNA levels, suggesting low mitochondrial damage (Extended Data Fig. 1j). Although HFCS feeding alone did not induce advanced stages of fibrosis detectable by histological analysis (Extended Data Fig. 1k), qPCR analysis indicated downregulated gene expression of HFCS-induced liver fibrosis markers by simultaneous or delayed inulin supplementation (Fig. 1l). Thus, even delayed inulin intake can reverse HFCS-induced systemic metabolic dysfunctions and liver damage.

### The effect of inulin on hepatic lipid synthesis and oxidation

**Fig. 2:**
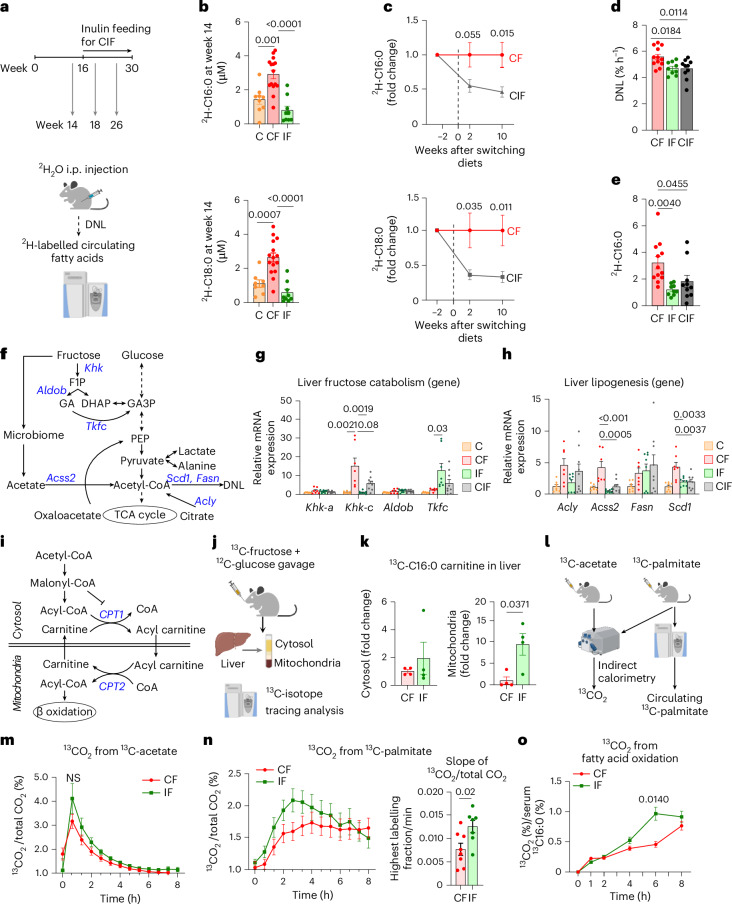
Inulin supplementation suppresses hepatic lipogenesis and increases FAO. **a**, Schematic of lipogenesis measurements using ^2^H_2_O tracing. i.p., intraperitoneal **b**, ^2^H-labelled fatty acids in circulating lipids on week 14 (*n* = 9, 16, 9 mice). **c**, ^2^H-labelled fatty acids in circulating lipids before and after switching diets for the CIF group. Data are fold changes relative to CF (*n* = 9, 9 mice). **d**, DNL rate measured by ^2^H_2_O tracing after normalization to body water enrichment (*n* = 12, 9, 10 mice). See Methods for more details. **e**, ^2^H-labelled palmitate concentration normalized to body water enrichment (*n* = 12, 10, 10 mice). **f**, Fructose catabolism and lipogenesis pathways. GA, glyceraldehyde; GA3P, glyceraldehyde-3-phosphate. PEP, phosphoenolpyruvate. **g**, Liver fructose catabolism gene expression (*n* = 8, 7, 8, 9 mice). **h**, Liver lipogenesis gene expression (*n* = 8, 7, 8, 9 mice). **i**, FAO pathway. **j**, Schematic of ^13^C-fructose tracing and hepatic cytosol and mitochondria fractionation. **k**, ^13^C-labelled C16:0 carnitine levels in cytosol versus mitochondria fraction of liver (*n* = 4, 4 mice). **l**, Schematic of whole-body fatty acid oxidation measurements using ^13^C-acetate and ^13^C-palmitate tracing. **m**, The ratio of ^13^CO_2_ to unlabelled CO_2_ over time after ^13^C-acetate administration (*n* = 8, 7 mice). **n**, The ratio of ^13^CO_2_ to unlabelled CO_2_ over time (left) and slope up to the maximum point (right) after ^13^C-palmitate administration (*n* = 8, 7 mice). **o**, ^13^CO_2_ from ^13^C-palmitate after normalization to circulating ^13^C-palmitate (*n* = 8, 7 mice). Data are means; error bars, s.e.m. *P* values determined by one-way ANOVA with Tukey’s HSD test (**b**,**d**–**h**), two-way ANOVA with Tukey’s HSD test (**m**,**o**) or two-sided unpaired Student’s *t*-test (**c**,**k**,**n**). NS, not significant. Illustrations in **a**, **j**, and **l** created with BioRender.com. Source data

In patients with MASLD, hepatic de novo lipogenesis (DNL) is a major contributor to steatosis development, and fructose is a potent DNL inducer^22,24,51,52^. Therefore, we sought to determine the effect of simultaneous and delayed inulin provision on hepatic DNL using a deuterated water (^2^H_2_O) tracer throughout the diet interventions (Fig. 2a). We first measured circulating levels of ^2^H-labelled saponified fatty acids that reflect hepatic DNL. Mice fed HFCS alone for 14 weeks showed significantly increased ^2^H-labelled fatty acids (Fig. 2b). Simultaneous or delayed inulin supplementation suppressed or reversed such induction (Fig. 2b,c). These results motivated us to quantitatively analyse hepatic DNL flux by calculating the appearance rate of ^2^H-labelled saponified palmitate per cent in blood after normalization to body water enrichment^53^ (Extended Data Fig. 1l). This analysis revealed that both simultaneous and delayed inulin supplementation significantly reduced HFCS-induced DNL (Fig. 2d). We also noticed a significant reduction both in circulating saponified palmitate following inulin supplementation (Extended Data Fig. 1m) and ^2^H-labelled palmitate levels normalized to body water enrichment (Fig. 2e). Considering that most circulating palmitate reflects triglycerides released from the liver, these data suggest that inulin not only decreases hepatic DNL but also affects other processes, such as hepatic secretion of triglycerides or clearance of circulating triglycerides.

Excessive fructose catabolism in the liver has been shown to drive the gene expression of DNL enzymes^22,23^. We therefore measured hepatic gene expression of enzymes for fructose catabolism and DNL (Fig. 2f). As previously reported^54^, HFCS feeding strongly induced the active isoform of *Khk* (*Khk-c*) (Fig. 2g). Simultaneous and delayed inulin supplementation blocked such induction (Fig. 2g). Intriguingly, *Tkfc*, an enzyme that converts fructose-derived toxic glyceraldehyde to glyceraldehyde-3-phosphate (Fig. 2f), was only induced when mice were fed both HFCS and inulin (Fig. 2g). Given that glyceraldehyde is a reactive metabolite that generates glycation products, which can damage DNA, RNA and proteins and induce hepatocyte cell death^55^, the induction of *Tkfc* expression by inulin may exert hepato-protective effects. In terms of DNL enzymes, HFCS induced gene expression of *Acly* (ATP citrate lyase), *Acss2* (acyl-CoA synthetase short-chain family member 2), *Fasn* (fatty acid synthase) and *Scd1* (stearoyl-coenzyme A desaturase 1) (Fig. 2h). Simultaneous and delayed inulin supplementation suppressed gene expression of *Acss2* and *Scd1* (Fig. 2h). Therefore, we conclude that inulin-induced mitigation or reversal of hepatic pathologies is in part attributed to the suppression of excessive hepatic fructose catabolism and DNL.

Next, we investigated the effect of inulin on fatty acid oxidation (FAO), which is normally suppressed by DNL through malonyl-CoA-mediated carnitine palmitoyltransferase 1 (CPT1) inhibition (Fig. 2i). Given the effect of inulin on suppressing DNL, we speculated that inulin activates FAO, thereby contributing to the reversal of HFCS-induced steatosis. During FAO, cytosolic fatty acids should be first conjugated with carnitine, forming acylcarnitine for their import into mitochondria (Fig. 2i). To measure newly synthesized acylcarnitine from fructose, we orally provided mice with a ^13^C-fructose tracer and fractionated the cytosol and mitochondria from fresh liver (Fig. 2j and Extended Data Fig. 1n,o). We found that inulin feeding significantly increased the levels of ^13^C-labelled acylcarnitine in the mitochondria but not in the cytosol (Fig. 2k). These data suggest that compared to HFCS feeding alone, HFCS with inulin feeding increased hepatic fructose carbon use to generate mitochondrial acylcarnitine for FAO.

To more quantitatively assess systemic FAO, we compared the oxidation of orally provided ^13^C-palmitate versus ^13^C-acetate (fully oxidizable) into ^13^CO_2_ using indirect calorimetry (Fig. 2l). This comparison is critical because CO_2_ fixation reactions (by pyruvate carboxylase, urea carboxylase and so forth) affect total ^13^CO_2_ exhalation^56,57^. We did not observe significant differences in ^13^CO_2_ production from ^13^C-acetate (Fig. 2m). By contrast, upon ^13^C-palmitate administration, inulin-fed mice showed accelerated exhalation of ^13^CO_2_ (Fig. 2n). Given that orally provided ^13^C-palmitate is mixed with unlabelled palmitate from triglycerides in blood, we measured blood ^13^C-palmitate enrichment and normalized ^13^CO_2_ production to estimate the total circulating palmitate oxidation (Extended Data Fig. 1p)^58^. Inulin-fed mice showed significantly increased ^13^CO_2_ production (Fig. 2o). These results suggest that inulin suppresses lipogenesis but boosts FAO, which contributes to the prevention or reversal of HFCS-induced hepatic steatosis.

### The impact of inulin on preventing fructose spillover to liver and colon

**Fig. 3:**
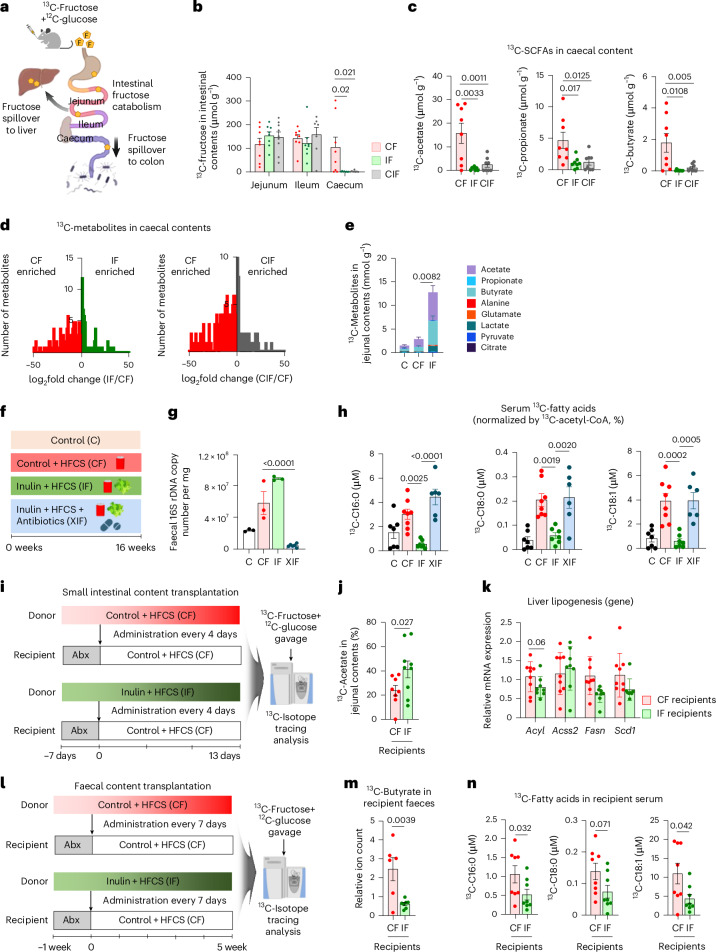
Inulin-fed small intestinal microbiome suppresses dietary fructose spillover. **a**, Schematic of dietary fructose catabolism by the host organs and gut microbiome. The small intestine first catabolizes fructose, and the leftover fructose spills over to the liver or colon and induces lipogenesis and gut dysbiosis. **b**, ^13^C-fructose levels in various intestinal contents 30 min after oral provision of HFCS with ^13^C-labelled fructose (*n* = 8, 8, 9 mice). **c**, ^13^C-labelled SCFAs in caecal contents (*n* = 8, 8, 8 mice). **d**, Comparison of labelled metabolite abundances in caecal contents between CF versus IF (left) (*n* = 8, 8 mice) or CF versus CIF (right) (*n* = 8, 8 mice). **e**, ^13^C-labelled metabolites in jejunal contents (*n* = 7, 7, 7 mice). **f**, Dietary intervention groups. XIF, antibiotics-treated group. **g**, Faecal 16S rDNA copy number (*n* = 3, 3, 3, 6 mice). **h**, ^13^C-labelled circulating saponified fatty acids normalized to hepatic ^13^C-acetyl CoA fraction, 1 h after provision of HFCS with ^13^C-labelled fructose (*n* = 7, 8, 8, 6 mice). **i**, Schematic of small intestinal microbiome transplantation experiments from donors (CF or IF) to recipients (CF after antibiotics). **j**,**k**, ^13^C-labelled acetate fraction in jejunal content (**j**) and liver lipogenesis gene expression (**k**) in recipient mice, 1 h after provision of HFCS with ^13^C-labelled fructose (*n* = 9, 9 mice). **l**, Schematic of faecal transplantation experiments. **m**,**n**, ^13^C-labelled butyrate in faeces (**m**) and ^13^C-labelled circulating saponified fatty acids (**n**) in recipient mice 1 h after provision of HFCS with ^13^C-labelled fructose (*n* = 8, 8 mice). Data are means; error bars, s.e.m. *P* values determined by one-way ANOVA with Tukey’s HSD test (**b**,**c**,**e**,**g**,**h**), two-sided unpaired Student’s *t*-test (**d**) or one-sided unpaired Student’s *t*-test (**j**,**k**,**m**,**n**). Illustrations in **a, i** and **j** created with BioRender.com. Source data

**Extended Data Fig. 2:**
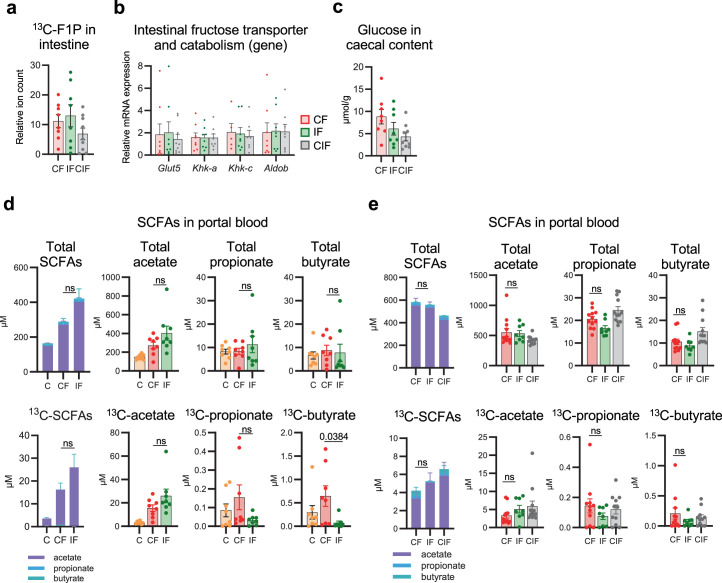
Inulin does not affect fructose catabolism by the host small intestine. **a**, ^13^C-labeled F1P abundances in small intestine, 30 min after oral provision of HFCS with fructose ^13^C-labeled (n = 8,8,9 mice). **b**, Small intestinal fructose transporter and catabolism gene expression (n = 8,8,9 mice). **c**, Glucose levels in caecal content, 30 min after oral provision of HFCS (n = 8,8,9 mice). **d-e**, Total and ^13^C-labeled SCFAs concentrations in portal blood, 1 h after oral provision of HFCS with fructose ^13^C-labeled (n = 7,7,7 mice, left), (n = 12,8,12 mice, right). Data are mean±s.e.m. P-values by one-way ANOVA with Tukey’s HSD test. ns=not significant. Source data

Previous studies have shown that fructose catabolism in the small intestine reduces fructose spillover to the liver and colonic microbiome, thereby suppressing hepatic DNL, liver steatosis and gut dysbiosis^14,15,59^. To determine how inulin affects small intestinal fructose catabolism and fructose spillover, we orally provided mice with a ^13^C-fructose tracer (with unlabelled glucose) (Fig. 3a). Inulin did not affect the production of labelled fructose 1-phosphate (Extended Data Fig. 2a), the metabolite marker of fructose catabolism (Fig. 2f), or the gene expression of fructose transporters and catabolic enzymes in the small intestine (Extended Data Fig. 2b). Moreover, inulin did not affect the amount of fructose reaching the small intestine, as shown by similar concentrations of ^13^C-fructose in the jejunal and ileal contents between groups (Fig. 3b). However, ^13^C-fructose was nearly absent in the caecum of inulin-fed mice (Fig. 3b). By comparison, glucose levels were similar in the caecum between groups (Extended Data Fig. 2c). These data suggest reduced fructose spillover to the colon in inulin-fed mice.

The absence of fructose in the caecum of inulin-fed mice can also reflect faster removal of fructose by the gut microbiome (thus, fructose disappeared quickly). To examine this possibility, we measured caecal labelled SCFAs, the most abundant metabolic products of microbial fructose catabolism^15^. Labelled SCFAs in caecal contents displayed similar depletion in inulin-fed mice (Fig. 3c), excluding the possibility of fast fructose catabolism by the colonic microbiome. Further supporting this notion, global untargeted metabolomics also revealed overall diminished fructose-derived labelled metabolites in the caecum of mice fed fructose and inulin (Fig. 3d). Therefore, we conclude that inulin supplementation blocks fructose spillover to the colon.

Interestingly, although fructose-derived SCFA production was reduced in the caecum of mice fed fructose and inulin (Fig. 3c), it was increased in the jejunal contents of the same mice, especially for acetate and butyrate (Fig. 3e). By comparison, neither labelled SCFAs nor total SCFAs were increased in the portal blood of mice fed both fructose and inulin from two independent mouse cohorts (Extended Data Fig. 2d,e). SCFAs such as butyrate have been suggested as host-beneficial products of microbial nutrient catabolism^60^. Although previous studies have shown an increase in portal SCFA levels after inulin feeding^61–63^, our data suggest that simultaneous feeding of fructose, which alters the host organs and intestinal microbial community^64,65^, appears to counteract this inulin effect. Together, these data suggest that inulin supplementation enhances fructose catabolism by the microbiome of the small intestine, without affecting the host’s small intestine fructose catabolism. This can lead to reduced fructose spillover to liver and colon. Given that inulin is a fructose polymer, gut microbiome using inulin as a carbon source may also boost fructose catabolism^66^, lowering the host’s exposure to dietary fructose.

### Gut microbiome mediates inulin’s effects on reducing fructose spillover

**Extended Data Fig. 3:**
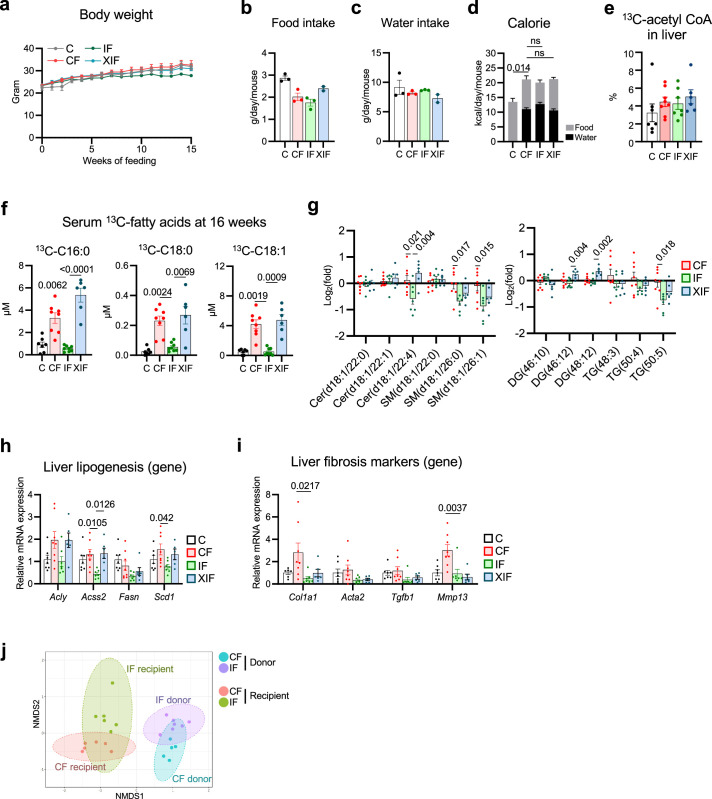
Antibiotics reverse inulin’s effects. **a**, Body weight (n = 7,8,7,6 mice). **b-d**, Daily food, water, and calorie intake (n = 3,3,3,2 cages). **e**, ^13^C-labeled acetyl CoA in liver (n = 7,8,7,6 mice), 1 hr after provision of HFCS with fructose ^13^C-labeled in mice fed the indicated diet for 16 weeks (n = 7,8,8,6 mice). **f**, ^13^C-labeled circulating saponified fatty acids. **g**, Abundances of the indicated hepatic lipid species normalized to the C group (n = 8,7,6 mice), **h-i**, Liver lipogenesis (**h**) and fibrosis marker (**i**) gene expression (n = 7,8,7,6 mice). **j**, NMDS plot showing microbial diversity among donor and recipient mice of jejunal microbiome transplant experiment (n = 5-7 mice/group). Data are mean±s.e.m. P-values determined by one-way ANOVA with Tukey’s HSD test. Source data

To determine whether the gut microbiome is critical for the effects of inulin on reducing fructose spillover, we treated mice fed both inulin and fructose with an antibiotic cocktail (XIF group) (Fig. 3f). Faecal 16S rDNA copy numbers confirmed successful microbiome depletion by antibiotics treatment (Fig. 3g), without affecting body weight gain, food, water or total calorie intake (Extended Data Fig. 3a–d). We first aimed to determine whether antibiotic treatment affects hepatic fructose carbon usage for fatty acid synthesis. After oral ^13^C-fructose provision, ^13^C enrichment of hepatic acetyl CoA, the primary precursor for fatty acid synthesis, was similar between the groups (Extended Data Fig. 3e). However, circulating ^13^C-labelled fatty acids normalized to hepatic acetyl CoA ^13^C enrichment revealed that antibiotics abolished inulin’s suppressive effect on fatty acid synthesis using fructose carbons (Fig. 3h and Extended Data Fig. 3f). Liver lipidomics and qPCR analysis also indicated that antibiotic treatment reversed the impact of inulin on decreasing liver lipid contents and gene expression of DNL enzymes and fibrosis markers (Extended Data Fig. 3g–i). These data suggest that the gut microbiome mediates the advantageous impacts of inulin.

We next conducted gut microbiota transplantation experiments to determine whether inulin-mediated effects are transmittable. Donor mice were fed HFCS alone (CF) or with inulin (IF) for 1 month to ensure the adaptation of the microbiome to each diet. In the meantime, recipient mice were fed only HFCS for 1 month to induce basal DNL. Then, after treatment of antibiotics to recipient mice for 1 week, the jejunal microbiome was transplanted from each donor group to recipients every 4 days for 13 days (Fig. 3i). After the fourth transplantation, we performed 16S rRNA sequencing in the small intestinal contents of the donor versus recipient mice to compare their gut microbiome profiles. Beta diversity comparison by non-metric multidimensional scaling (NMDS) based on the Bray-Curtis index showed that CF and IF recipients resemble their respective donors on NMDS2. On the other hand, NMDS1 separates donors from recipients, probably reflecting the antibiotics pre-treatment effects on recipients (Extended Data Fig. 3j). These microbiome analysis data suggest that our jejunal microbiome transplant was successful.

Importantly, ^13^C-fructose tracing revealed that IF recipients exhibited higher fructose catabolism in the jejunum than CF recipients (Fig. 3j). IF recipients also showed a trend of decreased lipogenic gene expression in the liver (Fig. 3k). Given that faecal transplant is more feasible for human applications, we also examined whether the effect of inulin is transmittable by faecal microbiome. We transplanted faecal microbiome from each donor group to recipients weekly for 5 weeks, followed by oral ^13^C-fructose tracing in recipient mice at the terminal endpoint (Fig. 3l). IF recipients exhibited substantially lowered fructose spillover to the colon compared to CF recipients (Fig. 3m). Concomitantly, IF recipients displayed lowered production of labelled fatty acids from fructose compared to CF recipients (Fig. 3n). Therefore, the effect of inulin on suppressing colonic fructose spillover and DNL (reflecting hepatic fructose spillover) is transferable by the gut microbiome.

### Hepatic metabolic rewiring by inulin under HFCS consumption

**Fig. 4:**
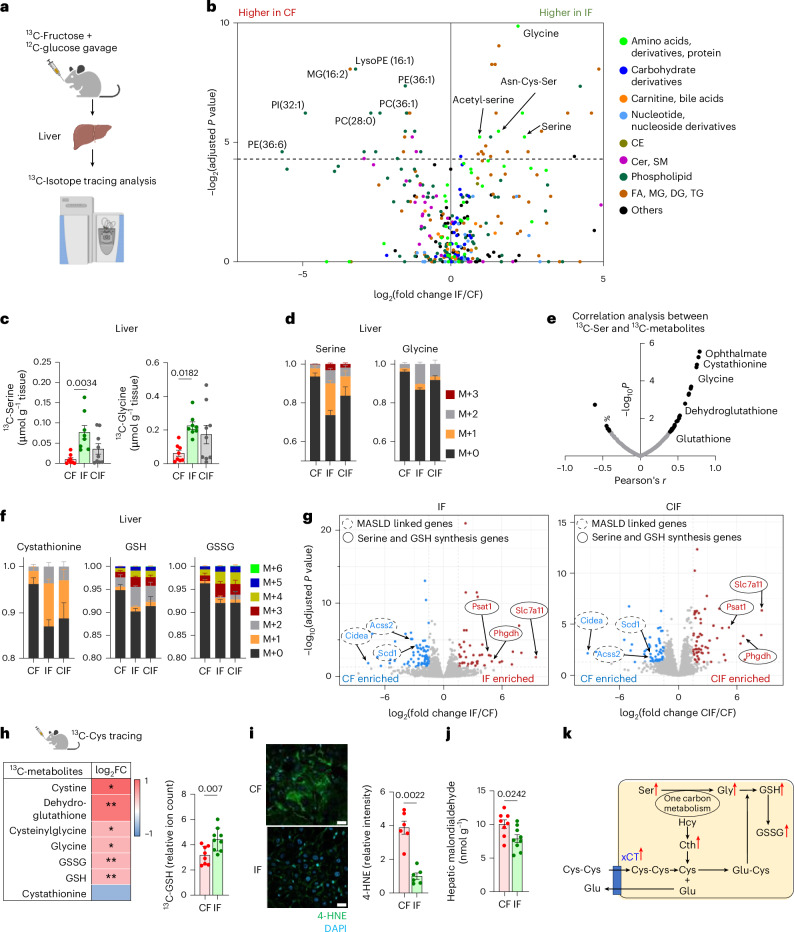
Inulin rewires hepatic fructose carbon use toward serine and GSH biosynthesis. **a**, Schematic of ^13^C-fructose tracing and untargeted metabolomics in liver. **b**, Comparison of hepatic ^13^C-labelling (%) of metabolites between IF and CF, 30 min after oral provision of HFCS with ^13^C-labelled fructose, by Student’s *t*-test followed by false discovery rate correction. Different colours indicate metabolite categories. CE, cholesteryl ester; FA, fatty acid; MG, monoacylglycerol (*n* = 8, 8 mice). **c**,**d**, ^13^C-labelled abundances (**c**) and labelling fractions (**d**) of serine and glycine in liver (*n* = 8, 8, 9 mice). M, isotopologue. **e**, Correlation coefficient (*r*) and *P* values between ^13^C-labelled serine ion count and the indicated labelled metabolite ion counts. **f**, ^13^C-labelled fractions of the indicated metabolites in liver. GSSG, oxidized GSH (*n* = 8, 8, 9 mice). **g**, Comparison of hepatic gene expression between CF and IF (left) (*n* = 5, 5 mice) or CF and CIF (right) (*n* = 5, 4 mice). **h**, Comparison of ^13^C-labelled ion counts of the indicated metabolites between CF and IF in liver, 30 min after oral provision of ^13^C-Cys. *P < 0.05, **P < 0.01 (*n* = 8, 9 mice). FC, fold change. **i**, Immunofluorescence staining and quantitation of liver 4-HNE, a lipid peroxidation marker. Scale bars, 20 μm (*n* = 6, 6 mice). **j**, Hepatic malondialdehyde levels measured by TBARS assay (*n* = 8, 9 mice). **k**, Schematic of the GSH synthesis pathway. Red arrows indicate that metabolites and *Slc7a11* gene (which encodes xCT) are significantly upregulated in IF compared to CF. Hcy, homocysteine; Cth, cystathionine. Data are means; error bars, s.e.m. *P* values determined by one-way ANOVA with Tukey’s HSD test (**c**), Student’s *t*-test followed by false discovery rate correction (**b**,**g**) or two-sided unpaired Student’s *t*-test (**h**–**j**). Illustrations in **a** and **h** were created with BioRender.com. Source data

Given that inulin-fed mice showed less fructose carbon usage for fatty acid synthesis^23^ (Fig. 3h), we were curious about the fate of fructose carbons in their livers. To answer this question, we provided ^13^C-fructose (with unlabelled glucose) and performed metabolomics-based unbiased analysis in the liver to survey metabolic products derived from fructose (Fig. 4a). Unexpectedly, mice fed HFCS with inulin exhibited high labelling of glycine, serine and several serine-containing metabolites from ^13^C-fructose (Fig. 4b and Supplementary Table 2). Likewise, delayed inulin supplementation also exhibited an increased trend of hepatic serine and glycine synthesis from fructose (Fig. 4c). The proportions of newly synthesized serine and glycine made from fructose in inulin-fed mouse livers were substantial, at ~30% and ~15%, respectively (Fig. 4d).

Next, we sought to determine the biological implications of activated serine and glycine synthesis in the livers of inulin-fed mice. To this end, we performed a correlation-based unbiased analysis to identify metabolites most significantly associated with increased serine synthesis. This analysis identified several metabolites in the glutathione (GSH) synthesis pathway (Fig. 4e). Indeed, liver cystathionine, GSH and oxidized GSH showed higher labelling from fructose in mice fed inulin with HFCS compared to mice fed HFCS alone (Fig. 4f).

**Extended Data Fig. 4:**
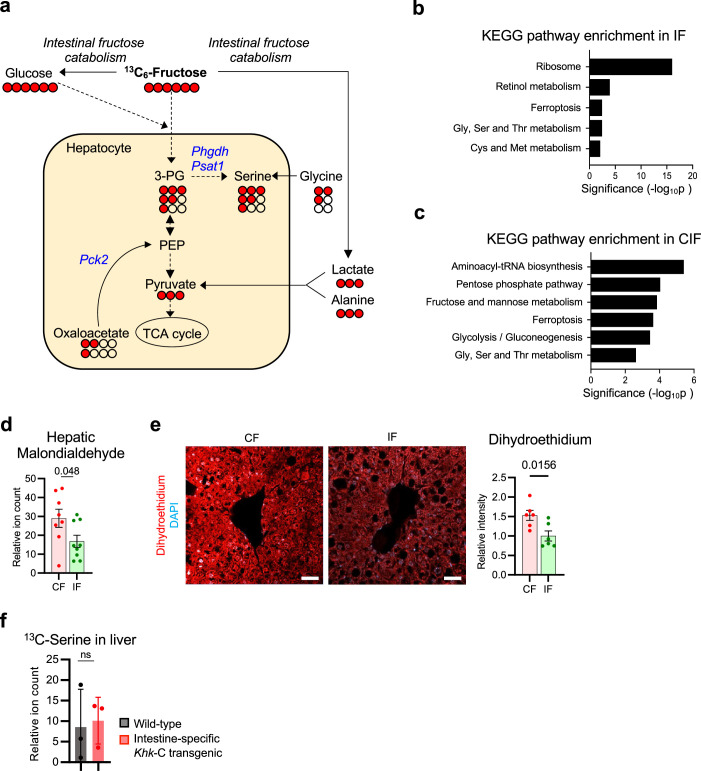
Inulin activates hepatic serine and GSH synthesis. **a**, Schematic of serine synthesis pathway from ^13^C-fructose. Red circles indicate labeled carbons. **b-c**, Kyoto Encyclopedia of Genes and Genomes (KEGG) pathway enrichment of IF(**b**) and CIF(**c**) compared CF (n = 5,5,4 mice for CF, IF and CIF groups). P-values by Fisher’s exact test. **d**, Hepatic malondialdehyde abundances measured by chemical derivatization and LC-MS measurement (n = 8,9 mice). **e**, Immunofluorescence staining and quantitation of liver dihydroethidium to measure ROS. Each dot indicates a mean of quadruplicate values from two independent experiments. Scale bars, 20 μm (n = 6,6 mice). **f**, ^13^C-labeled serine in liver of intestine-specific *Khk*-C transgenic mice, 1 hr after provision of HFCS with fructose ^13^C-labeled (n = 3, 3 mice). Data are mean±s.e.m. P-values by two-sided unpaired Student’s t-test. Source data

Serine and glycine can be de novo synthesized from the glycolytic intermediate, 3-phosphoglycerate, through *Phgdh* (3-phosphoglycerate dehydrogenase) and *Psat1* (phosphoserine aminotransferase 1)^67^ (Extended Data Fig. 4a). GSH, a tripeptide composed of glycine, glutamate and cysteine, requires both glycine synthesis and cystine uptake as the rate-limiting steps^68,69^. RNA sequencing (RNA-seq) revealed a drastic induction of cystine transporter *Slc7a11* (which encodes xCT) (~41-fold), *Phgdh* (~tenfold) and *Psat1* (~ninefold) in mice fed HFCS and inulin compared to mice fed HFCS alone (Fig. 4g and Supplementary Table 3). Delayed inulin supplementation exerted even more profound induction of *Slc7a11* (~53-fold), *Phgdh* (~22-fold) and *Psat1* (~11-fold) (Fig. 4g and Supplementary Table 3). Consistently, unsupervised pathway analysis captured glycine, serine and cysteine metabolism as one of the top upregulated pathways in mice fed HFCS with simultaneous or delayed inulin supplementation (Extended Data Fig. 4b,c). By contrast, MASLD-linked genes (including *Acss2* and *Scd1*) and *Cidea* (cell death-inducing DNA fragmentation factor) were decreased by inulin supplementation (Fig. 4g and Supplementary Table 3).

To directly examine GSH synthesis, we performed oral ^13^C-cysteine tracing experiments. The resulting data revealed enhanced hepatic synthesis of GSH and associated metabolites from cysteine in mice fed both inulin and HFCS compared to mice fed HFCS alone (Fig. 4h). GSH is critical for resolving oxidative stress and lipid peroxidation-mediated ferroptosis. Indeed, inulin-fed mice showed decreased hepatic lipid peroxidation markers, including 4-hydroxynonenal (4-HNE), malondialdehyde^70^ (Fig. 4i,j and Extended Data Fig. 4d) and dihydroethidium staining (Extended Data Fig. 4e). Therefore, we concluded that inulin supplementation activates both serine and glycine synthesis and cystine uptake in liver to augment GSH synthesis and mitigate HFCS-elicited hepatic lipid peroxidation (Fig. 4k).

### Inulin induces liver serine synthesis via the gut microbiome

**Fig. 5:**
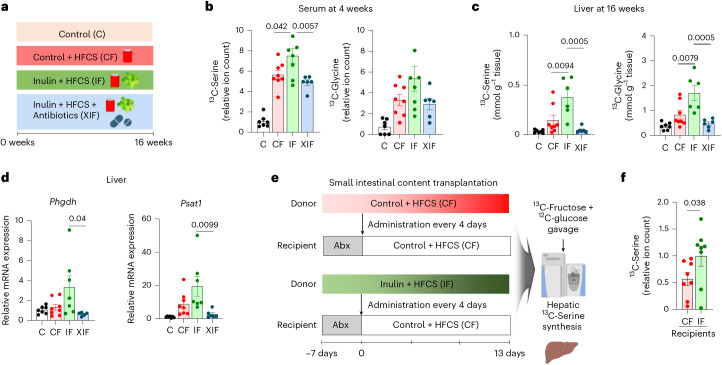
Inulin induces liver serine synthesis via gut microbiome. **a**, Experimental groups including the antibiotics-treated group (XIF). **b**,**c**, ^13^C-labelled abundances of serine and glycine in serum on week 4 (**b**) and liver on week 16 (**c**), 1 h after oral provision of HFCS with ^13^C-labelled fructose (*n* = 7, 8, 7, 6 mice). **d**, Serine synthesis gene expression in liver (*n* = 7, 8, 7, 6 mice). **e**, Schematic of jejunal microbiome transplantation experiments from donors (CF or IF) to recipients (CF after antibiotics). Abx, antibiotics. **f**, ^13^C-labelled serine abundances in liver of recipient mice (*n* = 8, 8 mice). Data are means; error bars, s.e.m. *P* values were determined by one-way ANOVA with Tukey’s HSD test (**b**–**d**) or one-sided unpaired Student’s *t*-test (**f**). Illustrations in **a** and **e** created with BioRender.com. Source data

We next sought to determine whether the effect of inulin on inducing liver serine synthesis is mediated by the gut microbiome. We performed ^13^C-fructose tracing after antibiotics treatment and measured liver serine synthesis (Fig. 5a). Inulin supplementation to HFCS-fed mice increased liver synthesis of serine and glycine using fructose carbons, but the effect was abolished by antibiotics (Fig. 5b,c). Moreover, antibiotics also blocked the inulin-elicited induction of serine biosynthesis genes *Phgdh* and *Psat1* (Fig. 5d).

To further determine whether the effect of inulin on liver serine synthesis is transmittable via the microbiome, we conducted small intestinal microbiota transplant experiments from donors fed HFCS with or without inulin to recipients fed HFCS alone following antibiotics treatment (Fig. 5e). ^13^C-fructose tracing at the end of transplantation cycles revealed that mice that received gut microbiome from the donor mice fed HFCS with inulin showed significantly higher serine synthesis than mice that received gut microbiome from the donor mice fed HFCS alone (Fig. 5f). Therefore, the gut microbiome is both necessary and sufficient for the inulin-induced serine synthesis.

We were curious whether inulin-mediated lipogenesis reduction and serine production are mechanistically linked. To test this idea, we implemented our previously reported intestine-specific *Khk*-C transgenic mice, which exhibit reduced hepatic lipogenesis owing to enhanced intestinal clearance of dietary fructose^16^. However, we found no increase in serine synthesis in these mice (Extended Data Fig. 4f), suggesting that these two biological processes (lipogenesis suppression and serine production induction by inulin) are driven through distinct mechanisms (for example, different gut microbiome species).

### *B.acidifaciens* contributes to inulin effects

**Fig. 6:**
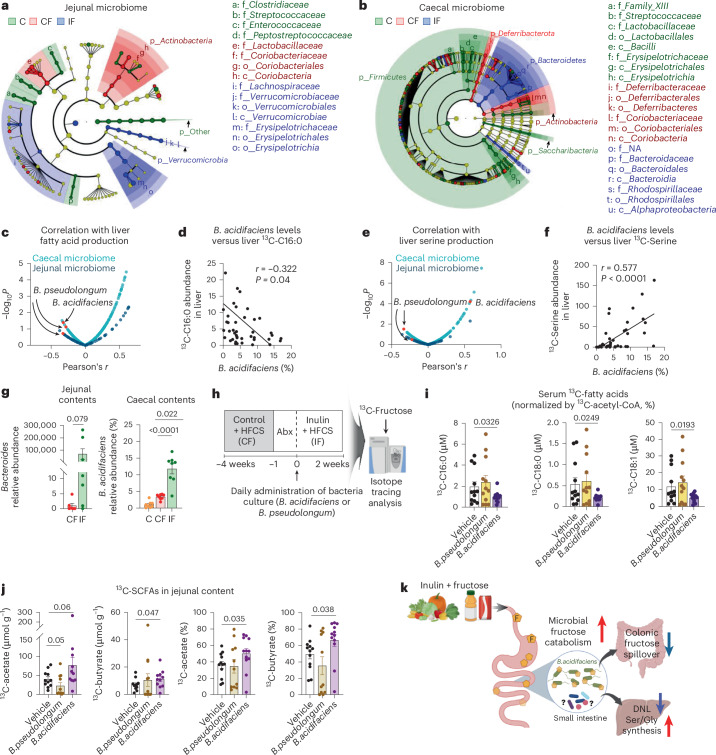
*B.* *acidifaciens* contributes to inulin’s effects on lipogenesis suppression and fructose catabolism in the small intestine. **a,b**, Linear discriminant analysis effect size analysis of jejunal (**a**) and caecal (**b**) microbial taxa. The cladogram shows the taxa with significant differences in abundance (from phylum to genus level) (*n* = 8, 8, 8 mice). **c**–**f**, Pearson correlation analysis between bacterial abundances and hepatic lipogenesis or serine synthesis. **g**, Relative abundance of *Bacteroides* spp. in jejunal contents (*n* = 6, 7 mice) and *B.* *acidifaciens* in caecal contents (*n* = 8, 8, 8 mice). **h**, Schematic of single-bacteria inoculation experiments. Recipient mice were fed HFCS alone for 4 weeks, followed by antibiotic treatment for 1 week. Anaerobically cultured bacteria were orally delivered to the recipient mice every day for 2 weeks while the mice received HFCS with inulin to promote bacteria survival and inulin usage. **i**, ^13^C-labelled circulating saponified fatty acids normalized to hepatic ^13^C-acetyl CoA fraction in the recipient mice, 1 h after oral provision of HFCS with ^13^C-labelled fructose (*n* = 12, 12, 12 mice). **j**, ^13^C-labelled concentrations (left two) and carbon fractions (right two) of the indicated SCFAs in the jejunal contents of the recipient mice, 1 h after oral provision of HFCS with ^13^C-labelled fructose (*n* = 12, 11, 12 mice). **k**, Proposed model of inulin’s multi-modal effects on stimulating small intestinal microbial breakdown of dietary fructose, reducing fructose spillover to colon, reducing hepatic lipogenesis and augmenting hepatic serine and GSH synthesis. Data are means; error bars, s.e.m. *P* values determined by one-way ANOVA with Tukey’s HSD test (**g**, right panel, **i**,**j**) or by one-sided unpaired Student’s *t*-test (**g**, left panel). Illustrations in **k** were created with BioRender.com. Source data

**Extended Data Fig. 5:**
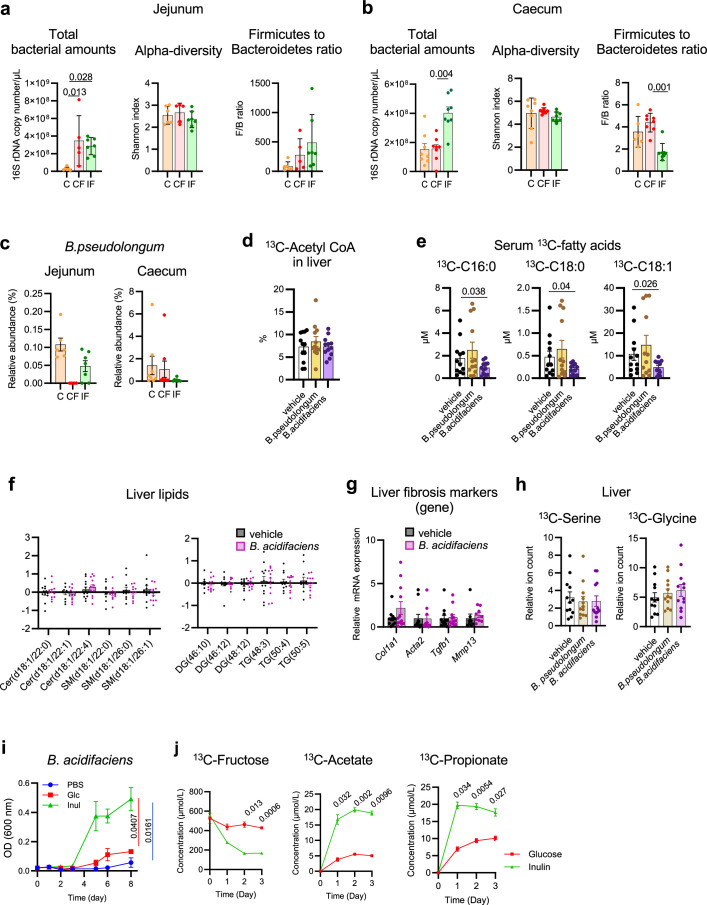
Characterization of gut microbiome in mice fed HFCS with or without inulin. **a**, Total bacterial amounts, Alpha-diversity, and Firmicutes to Bacteroidetes ratio in jejunum (n = 6,5,7 mice). **b**, Total bacterial amounts, Alpha-diversity, and Firmicutes to Bacteroidetes ratio in caecum (n = 8,8,8 mice). **c**, Relative abundance of *B. pseudolongum* in jejunal (n = 6,5,7 mice) and caecal content (n = 8,8,8 mice). **d**, ^13^C-labeled acetyl CoA enrichment (%) in liver, 1 hr after provision of HFCS with fructose ^13^C-labeled (n = 12,12,12 mice). **e**, ^13^C-labeled circulating saponified fatty acids (n = 12,12,12 mice). **f**, Relative abundances of the indicated hepatic lipid species (n = 12,12 mice). **g**, Gene expression of fibrosis markers in liver (n = 12,12 mice). **h**, Hepatic ^13^C-labeled serine and glycine abundances in mice that received vehicle or the indicated bacteria species, 1 hr after provision of HFCS with fructose ^13^C-labeled (n = 12,12,12 mice). **i**, Growth changes of *B. acidifaciens* in response to the addition of inulin or glucose (n = 3). **j**, Changes in ^13^C-fructose consumption and labeled short-chain fatty acid production of *B. acidifaciens* over incubation time (n = 3). Data are mean±s.e.m. P-values by one-way ANOVA with Tukey’s HSD test (**a-e, k**) or two-way ANOVA with Tukey’s HSD test (**i-j**). Source data

Finally, to identify gut microbiota species that mediate inulin’s effects, we performed 16S rRNA sequencing of the contents of both the small and large intestines of mice fed control water, HFCS alone or HFCS with inulin. Linear discriminant analysis effect size revealed marked differences in the microbial composition at each taxonomic level across the groups, with a maximum depth to genus level (Fig. 6a,b). As previously reported, inulin supplementation enriched *Bacteroidetes* in the large intestine, which is known to be the primary degrader of polysaccharides^71,72^. Interestingly, consumption of either HFCS alone or with inulin increased total bacterial contents in the small intestine (Extended Data Fig. 5a). Alpha diversity or the *Firmicutes* to *Bacteroidetes* ratio was not changed (Extended Data Fig. 5a). In the large intestine, compared to mice fed HFCS alone, mice fed both HFCS and inulin showed increased total bacterial contents, with no change in alpha diversity but a decreased *Firmicutes* to *Bacteroidetes* ratio (Extended Data Fig. 5b). This suggests improved gut microbiome health by inulin, given that an increased *Firmicutes* to *Bacteroidetes* ratio is a marker of microbiome dysbiosis in patients with metabolic disease^73–75^. Therefore, reduced fructose spillover by inulin-adapted small intestinal gut microbiome may contribute to a healthy microbiome in the large intestine.

From this global sequencing analysis, we next sought to identify the bacterial species contributing to inulin-induced metabolic changes in the liver. We performed a correlation-based unbiased analysis between the abundance of each bacterial species and hepatic DNL (Fig. 6c,d) or serine production (Fig. 6e,f). Although most bacterial species showed positive correlations with hepatic DNL (Fig. 6c), *B.* *acidifaciens* and *Bifidobacterium pseudolongum* exhibited a strong negative correlation with hepatic DNL (Fig. 6c,d), and *B. acidifaciens* showed a significant positive correlation with liver serine synthesis (Fig. 6e, f). Moreover, the abundance of *B.* *acidifaciens* was increased in both small and large intestines of mice fed HFCS and inulin (Fig. 6g)*. B.* *pseudolongum* did not show a clear pattern (Extended Data Fig. 5c).

To determine whether these candidate gut bacteria can recapitulate inulin’s effects, we performed single-bacteria inoculation experiments. Recipient mice were fed HFCS alone for 4 weeks to induce basal DNL, followed by antibiotics treatment for 1 week (Fig. 6h). Then, each anaerobically cultured bacteria or vehicle-alone control was orally delivered to the recipient mice every day for 2 weeks while the diet was switched to HFCS with inulin to promote not only the survival of the inoculated bacteria but also their inulin usage. On the last day, we performed ^13^C-fructose tracing. Measurements of circulating ^13^C-labelled fatty acids normalized to hepatic ^13^C enrichment of acetyl CoA (Extended Data Fig. 5d,e) revealed that treatment with *B.* *acidifaciens*, but not *B.* *pseudolongum* or vehicle treatment, significantly decreased fatty acid synthesis from fructose (Fig. 6i). Similarly, treatment with *B.* *acidifaciens*, but not *B.* *pseudolongum* or vehicle treatment, increased ^13^C-labelled acetate and butyrate in the contents of the small intestine (Fig. 6j), suggesting enhanced fructose catabolism by *B.* *acidifaciens* in the small intestine. However, *B.* *acidifaciens* treatment did not affect hepatic lipid accumulation, fibrosis or serine production (Extended Data Fig. 5f–h), suggesting that other bacterial species are involved or that the treatment duration was not sufficiently long.

Lastly, we investigated whether inulin supports the growth of *B.* *acidifaciens*. Indeed, inulin provision increased the growth of *B.* *acidifaciens* more than glucose (Extended Data Fig. 5i). Additionally, *B.* *acidifaciens* grown in inulin-containing media showed enhanced fructose catabolism and subsequent production of SCFAs (Extended Data Fig. 5j). Thus, inulin-adapted *B*. *acidifaciens* boosts fructose catabolism, which can mitigate the host’s exposure to excessive fructose. Altogether, our results support the conclusion that *B.* *acidifaciens* mediates, at least in part, inulin’s effect in suppressing HFCS-induced hepatic DNL by breaking down dietary fructose in the small intestine (Fig. 6k).

## Discussion

One of the clinical concerns of HFCS-induced pathologies is lean MASLD, which poses diagnostic challenges owing to the absence of significant weight gain. Lean patients with MASLD, therefore, have high risks of disease progression to MASH, cirrhosis and HCC without timely disease management. The causal mechanisms between excessive fructose consumption and lean MASLD have been extensively studied in animal models^16,48,76^. By contrast, studies on protective factors such as dietary fibres like inulin have been primarily performed in epidemiology or focused on the microbiome, specifically in the colon or faeces^77–79^. Upon investigating the interactions between these two common dietary components (fructose and inulin), we identified an unexpected microbe–host interaction mechanism by which inulin enhances fructose breakdown by the small intestinal microbiota (Fig. 6k). Even after hepatic steatosis has developed owing to HFCS through increased DNL and suppressed FAO via CPT1 (ref. ^80^), delayed inulin supplementation is sufficient to reverse it. Further human studies will be critical to determine the optimal amount of inulin in the diet and consumption durations that exhibit these protective effects.

Chemically, inulin is a soluble dietary fibre composed of one glucose molecule and numerous (~20–100) fructose molecules. Accordingly, when bacteria use inulin as a carbon source, they must activate enzyme machinery to break down fructose after cleaving inulin into monomeric fructose molecules. Indeed, in vitro cultured gut bacteria grown with inulin increase enzymes required for fructose degradation, such as β-fructofuranosidase, sucrose-6-phosphatase and phosphofructokinase^66,81^. Similarly, our in vivo and in vitro data suggest that when inulin-adapted gut microbiota encounter monomeric fructose, they effectively catabolize it, thereby reducing the host’s fructose exposure and mitigating metabolic consequences.

Through gut microbiome sequencing, in vivo isotope tracing and a correlation-based, unbiased approach, we identified *B.* *acidifaciens* as one of the contributors to these inulin-mediated effects. The abundance of *B.* *acidifaciens* was shown to be increased in the faeces of an inulin-fed MASH mouse model^31^. Our data suggest that *B.* *acidifaciens* also increases in both the small and large intestines after consumption of inulin and HFCS. This may indicate that *B.* *acidifaciens* can outgrow in the inulin-enriched intestinal microenvironment, even in the presence of incoming dietary HFCS. Intriguingly, *B.* *acidifaciens* has been found to prevent obesity in mice by promoting various hormones, including glucagon-like peptide 1 (GLP-1)^82^ and to alleviate concanavalin A-induced liver injury by blocking CD95 signalling^83^, although these studies have been performed in the absence of HFCS feeding. Our study suggests a mechanism by which inulin-adapted *B.* *acidifaciens* eliminates dietary fructose, thereby suppressing metabolic dysfunctions, regardless of obesity or exposure to hepatotoxic drugs.

In addition to fructose catabolism in the intestine, inulin supplementation also changes the fate of fructose carbons in the liver by redirecting the usage of fructose carbons to serine, glycine and GSH synthesis. Previous studies have observed lower glycine levels in individuals with MASLD^84,85^. In addition, depletion of glycine results in GSH deficiency, increasing susceptibility to oxidative stress and hepatic steatosis^86^. Given that hepatic glycine homoeostasis is regulated by *Shmt2* (serine hydroxymethyltransferase 2)^87^, these and other studies have focused on the role of *Shmt2* in MASLD^86–88^. Intriguingly, our RNA-seq data indicated that inulin supplementation does not affect the gene expression of *Shmt2*. Instead, inulin supplementation, in a microbiome-dependent manner, drastically increases *Phgdh* and *Psat1*, the key de novo serine synthesis pathway enzymes. Furthermore, inulin also activates transcription of *Slc7a11*, the cystine transporter, which can enhance GSH synthesis and block hepatic lipid peroxidation.

We then asked what mechanism might underlie serine biosynthesis induced by inulin. Decreased fructose spillover to the liver or lipogenesis is less likely to be the mechanism because intestine-specific *Khk*-C transgenic mice that exhibit lower fructose spillover and lipogenesis do not show increased serine synthesis (Extended Data Fig. 4f)^16^. Notably, antibiotics block inulin-induced serine synthesis, while *B.* *acidifaciens* suppresses lipogenesis without inducing serine synthesis. Therefore, other inulin-dependent microbiome species may signal to the liver to boost serine synthesis. Another remaining question surrounds the relative contribution of reduced lipogenesis and enhanced serine synthesis to inulin-mediated protective effects. Determining the contributions of each pathway would require future studies using genetically modified mouse models such as hepatocyte-specific *Phgdh* knockout mice.

Ultimately, it will be crucial to determine whether long-term provision of *B.* *acidifaciens* is sufficient to reverse hepatic steatosis and fibrosis and whether sexual dimorphism exists in inulin action, as our study used only males. To summarize, our findings on the effects of inulin in shifting fructose catabolism away from host organs and toward the small intestinal microbiome pave the way for protecting the host’s health by allowing the gut microbiome to consume toxic dietary nutrients.

## Methods

### Mouse studies

Animal studies followed protocols approved by the Institutional Animal Care and Use Committee of the University of California, Irvine (AUP-22-121). Male C57BL/6 mice (8 weeks old) were purchased from Jackson Laboratory. The duration of each experiment (for example, diet feeding) is indicated in the figure legends. In this study, only males were used because MASLD is more prevalent in men than women. Generation of *Khk*-C transgenic mice was previously reported in ref. ^16^. Mice were group-housed on a normal light–dark cycle (07:00–19:00 h) with free access to chow and water. We modified the open-source diet (Research Diets, D11112201) to reduce dextrose in the diet and replace corn starch with inulin. Mice were fed either a control (Research Diets, D21050401) or 10% (gm%) inulin diet (Research Diets, D21050402). The composition of both diets is shown in Supplementary Table 1. For HFCS provision, mice were provided either normal drinking water or 15% (weight/weight) fructose and 15% glucose mixture in drinking water. Animals were randomly assigned to experimental groups, and no specific method of randomization was used. No statistical methods were used to pre-determine sample sizes, but our sample sizes are similar to those reported in previous publications^15,16^. Unless otherwise indicated, experiments were replicated independently at least twice.

Daily water and food intake were determined by measuring the total consumption in a cage divided by the number of mice in the cage. For antibiotic treatment, a cocktail of antibiotics, including 0.5 g l^−1^ ampicillin, neomycin, metronidazole and 0.25 g l^−1^ vancomycin, was dissolved in HFCS-water. For gut bacteria transplantation, faecal or small intestinal contents (200 mg) were freshly collected from donor mice at 09:00–10:00 h using sterilized utensils and microcentrifuge tubes. The samples were immediately dissolved in 2 ml of sterilized anaerobic PBS containing 0.1 g l^−1^ of l-cysteine. The materials were then homogenized using sterilized pellet pestles and centrifuged at 500*g* for 3 min at 20 °C to remove particulate matter, based on studies by others that performed gut bacteria transplantation^89–91^. The resulting bacterial supernatant was supplemented with 10% sterilized glycerol and then transferred to antibiotic-treated recipient mice by oral gavage (200 µl per mouse) with a plastic feeding tube (Instech Laboratories) on the same day. The remaining solution was stored at −80 °C until use for oral gavage. Gut bacteria transplantation was performed weekly for 5 weeks for faeces (five times) and every 4 days for 13 days for jejunal contents (four times). For measurement of circulating levels of ^2^H-labelled fatty acid using ^2^H_2_O, mice received ^2^H_2_O dissolved in 0.9% NaCl by intraperitoneal injection (30 µl g^−1^) at 10:00 h. Mice were transferred to new cages without food. At 16:00 h, serum was collected by tail snip. For measurement of DNL flux, mice received ^2^H_2_O dissolved in 0.9% NaCl by intraperitoneal injection (30 µl g^−1^) at 18:00 h and serum was collected via tail snip at 09:00 h the following morning. For ^13^C-fructose tracing, mice received a solution of unlabelled glucose and ^13^C-fructose (2 g kg^−1^ body weight each) by oral gavage (10 µl g^−1^ body weight) at 09:00 h. For ^13^C-cysteine tracing, mice received a solution of 0.2 M ^13^C-cysteine by oral gavage (5 µl g^−1^ body weight) at 09:00 h. At 10:00 h, tail blood and tissues were collected. Intestine-specific *Khk*-C transgenic mice received a solution of unlabelled glucose and ^13^C-fructose (2 g kg^−1^ body weight each) by oral gavage (10 µl g^−1^ body weight) at 09:00 h, and liver tissues were collected at 10:00 h. Tail blood was collected by tail snip to measure circulating metabolites after different durations. Tissues were quickly dissected and snap-frozen in liquid nitrogen with a pre-cooled Wollenberger clamp. Multiple cohorts were used, and data were combined if there was no statistical difference between cohorts. For fasting glucose and insulin measurements, serum was collected after 10 h of fasting (08:00–18:00 h) by tail snip, glucose was measured using liquid chromatography–mass spectrometry (LC–MS) and insulin was measured using an Ultra Sensitive Mouse Insulin ELISA Kit (cat. no. 90080; Crystal Chem).

### Bacterial culture

*B.* *acidifaciens* (DSM 15896) was purchased from a German collection of microorganisms and cell cultures. *B.* *pseudolongum* (ATCC 25526) was obtained from American Type Culture Collection. *B.* *acidifaciens* was cultured on Columbia CNA agar medium containing sheep blood (Thermo Scientific) and chopped meat medium (CMM; Fisher Scientific) at 37 °C in the EZ Anaerobe Container System (BD). *B.* *pseudolongum* was cultured on modified BHI agar and broth (BD) at 37 °C in the EZ Anaerobe Container System (BD). To measure the growth of *B.* *acidifaciens* in response to inulin, PBS, 0.4 g l^−1^ glucose (Sigma-Aldrich) or 0.4 g l^−1^ inulin (Sigma-Aldrich) was added to 60% v/v CMM. *B.* *acidifaciens* was inoculated, and optical density at 600 nm was measured using a microplate reader (Victor). To measure the fructose catabolic activity of *B.* *acidifaciens*, the bacterium was cultured in CMM + glucose or CMM + inulin until the early stationary phase, washed with PBS and then the bacterial pellets were resuspended to the same optical density in CMM + glucose or CMM + inulin supplemented with 0.1 g l^−1^ ^13^C-fructose for a 3 day culture. To prepare a bacterial solution for oral delivery to mice, bacterial cultures were centrifuged and washed with PBS after 72 h of incubation. The bacterial pellets were resuspended in 25% (v/v) glycerol and stored at −80 °C before oral gavage to mice; 25% glycerol without any bacterium was used for the control. After HFCS provision and antibiotics treatment as described above, 200 µl of bacteria solution and glycerol were transferred to recipient mice by oral gavage (200 µl per mouse) with a plastic feeding tube (Instech Laboratories) every day for 2 weeks.

### Histology

Freshly collected liver tissues were fixed in 4% paraformaldehyde overnight, embedded in paraffin, sectioned and stained with haematoxylin and eosin (H&E). Tissues were submitted to the Experimental Tissue Shared Resource Facility at the University of California Irvine. For trichrome staining, Gomori’s Trichrome Stain Kit (Polysciences) was used. Images were captured with a high-resolution image scanner (Ventana DP200, Roche). Hepatic lipid accumulation was quantified by analysing the digital slides using QuPath (v.0.4.4)^92^. A pixel classifier was trained from representative images among the groups. This pixel classifier was then applied to annotations of the same size within each slide. These regions were measured, and the area values (in μm^2^) for the regions classified as lipids were used.

### Indirect calorimetry, ^13^C FAO analysis and echo magnetic resonance imaging

O_2_ consumption, CO_2_ release, respiratory exchange ratio, locomotor activity and heat production were monitored for individually housed mice using Phenomaster metabolic cages (TSE Systems). The climate chamber was set to 21 °C and 50% humidity, with a 12–12 h light–dark cycle (07:00–19:00 h) as the home-cage environment. Animals were entrained for 24 h in the metabolic cages before the start of each experiment to allow for environmental acclimation. Data were collected at 40 min intervals, and each cage was recorded for 3.25 min before time point collection. For measuring ^13^CO_2_, environmental levels of ^13^CO_2_ and total CO_2_ in the sealed cages were calibrated to ±1.1% ^13^CO_2_, as the natural abundance of ^13^C. Body composition was measured using an EchoMRI Whole Body Composition Analyzer, which provides whole-body fat and lean mass measurements. To account for potential changes in ^13^CO_2_ loss caused by CO_2_ fixation reactions (for example, those catalysed by pyruvate carboxylase or urea carboxylase)^56,57^, ^13^CO_2_ production was measured using indirect calorimetry after ^13^C-acetate administration. In brief, mice were fasted for 6 h and administered ^13^C-acetate by oral gavage (0.3 mg g^−1^ body weight), and ^13^CO_2_ recovery was measured for individually housed mice using Phenomaster metabolic cages (TSE Systems). Under the same conditions, we performed oral ^13^C-palmitate administration and ^13^CO_2_ measurements. To estimate the oxidation of the total circulating palmitate pool from circulating triglycerides and exogenous ^13^C-palmitate, we measured circulating un-esterified ^13^C-palmitate enrichment (%) in serial time points and used these values to normalize ^13^CO_2_ (ref. ^58^).

### Quantitative PCR with reverse transcription

RNA samples were prepared using TRIzol Reagent (Invitrogen) according to the manufacturer’s instructions. RNA was reverse-transcribed to cDNA using the iScript kit (Bio-Rad). The resulting cDNA was analysed by qPCR with reverse transcription using SYBR green master mix (Life Technologies) on a QuantStudio6 Real-Time PCR system (Life Technologies). Relative mRNA expression was calculated from the comparative threshold cycle values relative to housekeeping genes *Actin*, *36b4* and *Tbp*. Primer sequences were: Tgfb1 (forward, CTCCCGTGGCTTCTAGTGC; reverse, GCCTTAGTTTGGACAGGATCTG), Acta2 (forward, ATGCTCCCAGGGCTGTTTTCCCAT; reverse, GTGGTGCCAGATCTTTTCCATGTCG), Vim (forward, TTTCTCTGCCTCTGCCAAC; reverse, TCTCATTGATCACCTGTCCATC), Col1a1 (forward, GCTCCTCTTAGGGGCCACT; reverse, CCACGTCTCACCATTGGGG), Mmp13 (forward, CTTCTTCTTGTTGAGCTGGACTC; reverse, CTGTGGAGGTCACTGTAGACT), Glut5 (forward, TCTTTGTGGTAGAGCTTTGGG; reverse, GACAATGACACAGACAATGCTG), Khk-a (forward, TTGCCGATTTTGTCCTGGAT; reverse, CCTCGGTCTGAAGGACCACAT), Khk-c (forward, TGGCAGAGCCAGGGAGAT; reverse, ATCTGGCAGGTTCGTGTCGTA), Aldob (forward, CACCGATTTCCAGCCCTC; reverse, GTTCTCCACCTTTATCCTTTGC), Tkfc (forward, GCATCTCAGAGCAGAAGTGTG; reverse, CAAGTCAGGGTTAGAGGCTAC), Acly (forward, CAGCCAAGGCAATTTCAGAGC; reverse, CTCGACGTTTGATTAACTGGTCT), Acss2 (forward, ATGGGCGGAATGGTCTCTTTC; reverse, TGGGGACCTTGTCTTCATCAT), Fasn (forward, GGAGGTGGTGATAGCCGGTAT; reverse, TGGGTAATCCATAGAGCCCAG), Scd1 (forward, TTCAGAAACACATGCTGATCCTCATAATTCCC; reverse, ATTAAGCACCACAGCATATCGCAAGAAAGT), Tbp (forward, CCCTATCACTCCTGCCACACCAGC; reverse, GTGCAATGGTCTTTAGGTCAAGTTTAC), Actin (forward, CCCTGTATGCTCTGGTCGTACCAC; reverse, GCCAGCCAGGTCCAGACGCAGGATG) and 36b4 (forward, GGAGCCAGCGAGGCCACACTGCTG; reverse, CTGGCCACGTTGCGGACACCCTCC).

#### Mitochondria and cytosolic fractionation

To isolate liver mitochondria^93^, freshly isolated mouse liver was weighed and rapidly extracted and homogenized in ice-cold liver homogenization buffer (1 ml per 200 mg of liver; 200 mM sucrose, 5 mM Tris, 1 mM EGTA, 100 μg ml^−1^ digitonin pH 7.4), followed by centrifugation at 1000*g* for 1 min at 4 °C. The resulting 200 μl supernatants were combined with 500 μl spin buffer (150 mM sucrose, 5 mM Tris, 1 mM EGTA, 25 mM ammonium bicarbonate pH 7.4) to reduce suspension density. Each 700 μl mixture was carefully layered above 300 μl liver oil mix (60:40 silicone oil to dioctyl phthalate; density, 1.066 g ml^−1^ at 20–23.5 °C) and 100 μl of 23% glycerol in pre-mixed tubes, then centrifuged at 9,727*g* for 1 min at 4 °C. The cytosolic layer and most of the oil were aspirated, and the remaining oil was removed after freezing the lower layer in a dry ice–ethanol bath and washing with dry ice–cold hexane. The mitochondrial pellets were pooled to obtain the final sample.

#### Mitochondrial DNA analysis

For mtDNA copy number analysis, 50 ng of DNA was extracted with the Purelink Genomic DNA Mini (Invitrogen), and qPCR was performed with a pair of primers for mtDNA (MT-16S rRNA-ND1) with 18S for normalization. The following primers were used: MT-16S-ND1 (forward, CACCCAAGAACAGGGTTTGT; reverse, TGGCCATGGGTATGTTGTTAA) and 18S (forward, TAGAGGGACAAGTGGCGTTC; reverse, CGCTGAGCCAGTCAGTGT).

### RNA-seq analysis

RNA samples were prepared using TRIzol Reagent (Invitrogen) according to the manufacturer’s instructions. RNA concentration was quantified using fluorimetry (Qubit 2.0 fluorometer; Life Technologies), and quality was assessed using an Agilent BioAnalyzer 2100 (Agilent Technologies). Ribosomal RNA depletion and sample library preparation were performed using the Illumina TruSeq Stranded Total RNA with RiboZero. The libraries were then sequenced on a NovaSeq 6000 (Illumina) using paired-end sequencing. The quality of the raw sequencing data was assessed using FastQC, and all samples passed the quality control analysis. Raw sequencing reads were aligned to the mouse reference genome mm10 (GRCm38) using STAR, and RNA-seq counts were obtained using featureCounts. These raw counts were further analysed using DESeq2 for differential gene expression analysis. To elucidate the biological pathways involved in our study, we used Kyoto Encyclopedia of Genes and Genomes pathway enrichment analysis using Fisher’s exact test. For gene-set enrichment analysis, gene sets from the Molecular Signatures Database (v.2023.2) were used.

### Metabolite measurements using LC–MS

For aqueous metabolites extraction, serum (5 µl) was mixed with 150 µl of extraction solvent (40:40:20 methanol:acetonitrile:water, v:v:v) at −20 °C, vortexed and immediately centrifuged at 16,000*g* for 10 min at 4 °C. The supernatant (70 µl) was collected for LC–MS analysis. Frozen tissue samples were ground at liquid nitrogen temperature with a CryoMill (Retsch). The resulting tissue powder (approximately 20 mg) was weighed and then mixed with −20 °C extraction solvent containing 0.5% formic acid (40 µl per mg tissue), vortexed and neutralized with 15% NH_4_HCO_3_ (3.5 µl per mg tissue). Following vortexing and centrifugation at 16,000*g* for 10 min at 4 °C, the supernatant (70 µl) was loaded into LC–MS vials. Metabolites were analysed by a quadrupole–orbitrap mass spectrometer (Q-Exactive Plus Hybrid Quadrupole–Orbitrap, Thermo Fisher) coupled to hydrophilic interaction chromatography by heated electrospray ionization. LC separation was performed on an Xbridge BEH amide column (2.1 mm × 150 mm, 2.5 µm particle size, 130 Å pore size; Waters) at 25 °C using a gradient of solvent A (5% acetonitrile in water with 20 mM ammonium acetate and 20 mM ammonium hydroxide) and solvent B (100% acetonitrile). The flow rate was 150 µl min^−1^. The LC gradient was: 0 min, 90% B; 2 min, 90% B; 3 min, 75% B; 7 min, 75% B; 8 min, 70% B; 9 min, 70% B; 10 min, 50% B; 12 min, 50% B; 13 min, 25% B; 14 min, 20% B; 15 min, 20% B; 16 min, 0% B; 20.5 min, 0% B; 21 min, 90% B; and 25 min, 90% B. Autosampler temperature was set at 4 °C and the injection volume of the sample was 3 μl. MS analysis was acquired in negative and positive ion modes with Full MS scan mode from *m*/*z* 70 to 830 and 140,000 resolution with the following operational parameters: AGC target, 3 × 10^6^; maximum IT, 500 ms; sheath gas flow rate, 40; aux gas flow rate, 10; sweep gas flow rate, 2; spray voltage, +3.8 kV and −3.5 kV; spray current, 33 μA; capillary temperature, 300 °C; s-lens RF level, 50; aux gas heater temperature, 360 °C. MS2 analysis was acquired in negative and positive ion modes with Full MS/dd-MS2 from *m*/*z* 70 to 830. For full MS, the parameters were: resolution, 70,000; AGC target, 1 × 10^6^; maximum IT, 200 ms. For MS/dd-MS2, the parameters were: resolution, 17,500; AGC target, 1 × 10^5^; maximum IT, 50 ms; loop count, 15; isolation window, 1.2 *m*/*z*; stepped CE, ±20 and 50 eV with the following operational parameters: sheath gas flow rate, 40; aux gas flow rate, 10; sweep gas flow rate, 2; spray voltage, +3.8 kV and −3.5 kV; spray current, 33 μA; capillary temperature, 300 °C; s-lens RF level, 50; aux gas heater temperature, 360 °C. Data were analysed using the EI-MAVEN software and Compound Discoverer software (Thermofisher Scientific). The identity of metabolites was confirmed based on the retention time and accurate *m*/*z* of authentic synthesized chemical standards from Sigma-Aldrich, as well as MS2 fragmentation patterns available in HMDB (https://hmdb.ca) and mzCloud database (https://www.mzcloud.org). Natural isotope correction was performed with AccuCor2 R code (https://github.com/wangyujue23/AccuCor2). Labelled ion counts refer to the sum of all labelled forms, in which each form is weighted by the fraction of carbon atoms labelled. It is used to calculate fractional carbon labelling (%) by normalizing against the ion count of the total pool. The concentrations of selected metabolites were determined by calibration curves using authentic synthesized standards. The metabolite concentrations in tissues or intestinal contents were calculated using the following equation: concentration (μmol g^−1^) = concentration of extracted sample (μM) × volume of extraction solution (μl) / tissue weight (mg).

### Lipid measurements using LC–MS

For lipid extraction, samples were mixed with −20 °C isopropanol (150 µl per 5 µl serum and 40 µl per mg tissue), vortexed and immediately centrifuged at 16,000*g* for 10 min at 4 °C^15^. The supernatant (70 µl) was loaded into LC–MS vials. Lipids were analysed by a quadrupole–orbitrap mass spectrometer (Q-Exactive Plus Hybrid Quadrupole–Orbitrap) coupled to reverse-phase chromatography with electrospray ionization. LC separation was on an Atlantis T3 Column (2.1 mm × 150 mm, 3 µm particle size, 100 Å pore size; Waters) at 45 °C using a gradient of solvent A (1 mM ammonium acetate, 35 mM acetic acid in 90:10 water:methanol) and solvent B (1 mM ammonium acetate, 35 mM acetic acid in 98:2 isopropanol:methanol). The flow rate was 150 µl min^−1^. The LC gradient was: 0 min, 25% B; 2 min, 25% B; 5.5 min, 65% B; 12.5 min, 100% B; 16.5 min, 100% B; 17 min, 25% B; and 30 min, 25% B. MS analysis was acquired in positive ion mode with Full MS and MS/dd-MS2 scan mode from *m*/*z* 290 to 1,200. The MS operational parameters and data analysis are the same as for the metabolite analysis.

### Saponified fatty acid measurement using LC–MS

Serum (5 µl) or liver powder (20 mg) was incubated with 0.5 ml of 0.3 M KOH in 90% methanol at 80 °C for 1 h in a 2 ml glass vial. Then, formic acid (50 µl) was added for neutralization. The saponified fatty acids were extracted by adding 500 µl of hexane and vortexing. After 5 min for separation of the layers, 250 µl of the top hexane layer was transferred to a new glass vial. Samples were then dried under a nitrogen gas stream and redissolved in 100 µl (for serum) or 500 µl (for liver) of 1:1 isopropanol:methanol for LC–MS analysis^15^. Fatty acids were analysed by a quadrupole–orbitrap mass spectrometer (Q-Exactive Plus Hybrid Quadrupole–Orbitrap) coupled with reverse-phase chromatography with electrospray ionization. LC separation was performed on an Atlantis T3 Column (2.1 mm × 150 mm, 3 µm particle size, 100 Å pore size; Waters) at 45 °C using a gradient of solvent A (1 mM ammonium acetate, 35 mM acetic acid in 90:10 water:methanol) and solvent B (1 mM ammonium acetate, 35 mM acetic acid in 98:2 isopropanol:methanol). The flow rate was 150 µl min^−1^. The LC gradient was: 0 min, 25% B; 2 min, 65% B; 5.5 min, 100% B; 16.5 min, 100% B; 16.5 min, 25% B with a flow rate of 200 µl min^−1^; 19 min, 25% B with a flow rate of 200 µl min^−1^; 19.1 min, 25% B with a flow rate of 150 µl min^−1^; and 20 min, 25% B. MS analysis was acquired in negative ion mode with Full MS scan mode from *m*/*z* 200 to 530. The MS operational parameters and data analysis are the same as for the metabolite analysis

### Body water enrichment and DNL calculation

To quantify body water enrichment, 5 μl of serum, 5 μl of water, 4 μl of 1 M sodium hydroxide and 10 μl of acetone were mixed in a glass vial and incubated overnight at room temperature to promote ^2^H exchange between ^2^H_2_O in serum and acetone^94^. The resulting ^2^H-acetone was derivatized to 2,4-dinitrophenylhydrazine (2,4-DNPH)^95^. The 2,4-DNPH solution was prepared by dissolving 20 mg of 2,4-DNPH in 10 ml of ethanol with 100 μl of H_2_SO_4_ and 150 μl of water. The precipitate formed upon mixing was then isolated by filtration (cat. no. 09-790-D; Fisher Scientific). The filtrate was treated with 1 ml of H_2_SO_4_, and 5 μl of this solution was mixed with 20 μl of the sample solution. After a 2 h incubation at room temperature, 200 μl of ethanol was added, and the samples were transferred to 1.5 ml tubes for centrifugation at 4 °C for 20 min. The clear supernatant was transferred into a glass vial for LC–MS analysis. ^2^H_1_ acetone and unlabelled acetone were measured by LC–MS analysis, using the same method as for SCFA analysis (see below), and were used to calculate the fraction of ^2^H_1_ acetone. Calibration standards of known ^2^H fraction water were prepared by mixing naturally labelled water and 99.9% ^2^H_2_O. The ^2^H_1_ acetone fraction of ^2^H_2_O serial dilution in naturally labelled water was used to generate a standard curve. Then, the ^2^H_1_ acetone fraction in the serum samples was substituted into the standard curve equation to calculate body water enrichment, as previously described^53,96^. The contribution of fatty acid synthesis was determined using equation (1).

1 $$\mathrm{DNL} = \frac{{2\atop}\mathrm{H}\text{-labeled palmitate enrichment}}{\text{body water enrichment} \times \text{number of exchangeable hydrogens}}$$

^2^H-labelled palmitate enrichment was calculated using equation (2), where ^2^H_1_, ^2^H_2_ and ^2^H_3_ indicate the ^2^H-labelled fraction of each isotopologue.

2 $${\scriptstyle{2}\atop}{\rm{H}}\,{\rm{enrichment}}={\scriptstyle{2}\atop}{\rm{H}}_{1}+\left({\scriptstyle{2}\atop}{\rm{H}}_{2}\,{\times}\,2\right)+\left({\scriptstyle{2}\atop}{\rm{H}}_{3}\,{\times}\,3\right)$$

The number of exchangeable hydrogens (*n*) was calculated using the fractions of ^2^H_1_ and ^2^H_2_ palmitate as in equation (3). The rate of DNL per hour was determined by dividing the time elapsed since ^2^H_2_O administration.

3 $$\frac{{\scriptstyle{2}\atop}{\rm{H}}_{2}}{{2\atop}{\rm{H}}_{1}}=\frac{(n-1)}{2}\times \frac{{\rm{body}}\,{\rm{water}}\,{\rm{fraction}}}{(1-{\rm{body}}\,{\rm{water}}\,{\rm{fraction}})}$$

### SCFA measurement using LC–MS

Serum (5 µl) or intestinal content (1 mg) was mixed with derivatizing reagent (100 µl) and incubated for 1 h at 4 °C. The derivatizing reagent was prepared by mixing 12 mM *N*-(3-dimethylaminopropyl)-*N*′-ethylcarbodiimide, 25 mM 3-nitrophenylhydrazine and pyridine (4% v/v) in 40:40:20 methanol:acetonitrile:water (v/v/v). Samples were centrifuged at 16,000*g* for 10 min at 4 °C, and 10 µl of supernatant was mixed with 90 µl of the quenching reagent (0.5 mM β-mercaptoethanol in water). After centrifugation at 16,000*g* for 10 min at 4 °C, the supernatants were collected for LC–MS analysis. SCFAs were analysed using a quadrupole–orbitrap mass spectrometer (Q-Exactive Plus Hybrid Quadrupole–Orbitrap) coupled with reverse-phase chromatography with electrospray ionization. LC separation was performed on an Atlantis T3 Column (2.1 mm × 50 mm, 3 µm particle size, 100 Å pore size; Waters) using a gradient of solvent A (water) and solvent B (methanol) at 60 °C. The flow rate was 300 µl min^−1^. The LC gradient was: 0 min, 10% B; 2.3 min, 80% B; 3.6 min, 80% B; 3.7 min, 10% B; and 5 min, 10% B. MS analysis was acquired in negative ion mode with Full MS scan mode from *m*/*z* 100 to 300. The MS operational parameters and data analysis are the same as for the metabolite analysis.

### Malondialdehyde measurement using LC–MS

Malondialdehyde in liver tissue (10 mg) was mixed with 20 µl of butylated hydroxytoluene solution (1 g l^−1^ in ethanol) and 220 µl of 50% ethanol solution^97^. Samples were centrifuged at 16,000*g* for 10 min at 4 °C, and 200 µl of supernatant was mixed with 200 µl of 2,4-dinitrophenyl-hydrazine solution (0.05 M in acetonitrile:acetic acid 9:1 (v/v)). After incubating samples for 2 h at 60 °C, the samples were mixed with 530 µl of water and 1 ml of hexane. Samples were vortexed and incubated for 10 min at 25 °C to separate the layers. Then, 500 µl of the top hexane layer was transferred to a new glass vial. Samples were then dried under a nitrogen gas stream and redissolved in 200 µl of acetic acid solution (0.03% in acetonitrile:water 4:6 (v/v)). After centrifugation at 16,000*g* for 10 min at 4 °C, 150 µl of the supernatant was collected for LC–MS analysis. Malondialdehyde was analysed using a quadrupole–orbitrap mass spectrometer (Q-Exactive Plus Hybrid Quadrupole–Orbitrap) coupled to reverse-phase chromatography with electrospray ionization. LC separation was performed on an Atlantis T3 Column (2.1 mm × 150 mm, 3 µm particle size, 100 Å pore size; Waters) at 45 °C using a gradient of solvent A (1 mM ammonium acetate, 35 mM acetic acid in 90:10 water:methanol) and solvent B (1 mM ammonium acetate, 35 mM acetic acid in 98:2 isopropanol:methanol). The flow rate was 150 µl min^−1^. The LC gradient was: 0 min, 25% B; 2 min, 100% B; 5 min, 100% B; 11.5 min, 100% B; 11.6 min, 25% B; and 15 min, 25% B. MS analysis was acquired in negative ion mode with Full MS scan mode from *m*/*z* 200 to 400. The MS operational parameters and data analysis are the same as for the metabolite analysis. Malondialdehyde was also quantified using a thiobarbituric acid reactive substances (TBARS) assay kit (10009055, Cayman Chemical).

### Immunofluorescence imaging

Tissue samples were fixed in 4% buffered paraformaldehyde for 1 h at room temperature. After fixation, tissues were dehydrated with 30% sucrose in PBS overnight, frozen and embedded in Frozen Section Media (Leica) and cut into 20 μm-thick sections using a Cryocut Microtome (Leica). The samples were then blocked in protein block serum (Agilent) with 0.3% Triton X-100 (Thermo Fisher) for 1 h. For 4-HNE staining, the sections were incubated overnight at 4 °C with anti-4-HNE antibody (clone 12F7; Invitrogen, 1:200) in antibody diluent (Agilent), then with anti-mouse secondary antibody conjugated to Alexa Fluor 488 (1:1,000; Jackson ImmunoResearch) for 2 h at room temperature. For reactive oxygen species staining, the sections were incubated with 5 µM dihydroethidium (309800, Sigma-Aldrich) for 30 min at 37 °C. After staining, the samples were washed three times for 10 m each in PBS with 0.3% Triton X-100. Finally, tissue sections were mounted with Vectashield plus DAPI (4′,6-diamidino-2-phenylindole) (Vector Labs) and imaged with an LSM 980 confocal microscope (Zeiss).

### Bacteria 16S rDNA quantification

Bacterial DNA was extracted from faecal pellets (10–20 mg) using Quick-DNA Faecal/Soil Microbe Kits (Zymo Research) according to the manufacturer’s instructions. Purified DNA was amplified by qPCR using SYBR green master mix (Life Technologies) on a QuantStudio6 Real-Time PCR system (Life Technologies). DNA from the *E.* *coli* DH5a strain was used as a standard for determining the copy number of the 16S rDNA gene of universal bacteria by qPCR. Primer pairs targeting the bacterial universal 16S rRNA gene, *Bacteroides* spp. and *B.* *pseudolongum* were selected from previous studies^98,99^ as follows: bacterial universal 16S rRNA gene (forward, GTGGTGCACGGCTGTCGTCA; reverse, ACGTCATCCACACCTTCCTC), *B.* *pseudolongum* (forward, CRATYGTCAAGGAACTYGTGGCCT; reverse, GCTGCGAMGAKACCTTGCCGCT) and *Bacteroides* spp. (forward, CTGAACCAGCCAAGTAGCG; reverse, CCGCAAACTTTCACAACTGACTTA). Relative bacterial abundances of *Bacteroides* spp. and *B.* *pseudolongum* were calculated from threshold cycle values relative to universal bacterial abundance in each sample.

### 16S rRNA gene amplicon sequencing and analysis

The ZymoBIOMICS-96 MagBead DNA Kit (Zymo Research) was used to extract DNA from mouse jejunal or caecal contents. Bacterial 16S ribosomal RNA gene-targeted sequencing was performed using the Quick-16S NGS Library Prep Kit (Zymo Research). The bacterial 16S primers amplified the V3–V4 region of the 16S rRNA gene. The sequencing library was prepared using an innovative library preparation process in which PCR reactions were performed in real-time PCR machines to control cycles and, therefore, limit PCR chimera formation. The final PCR products were quantified with qPCR fluorescence readings and pooled together based on equal molarity. The final pooled library was cleaned with the Select-a-Size DNA Clean & Concentrator (Zymo Research), then quantified with TapeStation (Agilent Technologies) and Qubit (Thermo Fisher Scientific). The final library was sequenced on Illumina MiSeq with a v3 reagent kit (600 cycles). The sequencing was performed with 10% PhiX spike-in. Unique amplicon sequence variants were inferred from raw reads using the DADA2 pipeline. Taxonomy assignment was performed using Uclust from Qiime (v.1.9.1) with the Zymo Research 16S reference database. Composition visualization, alpha diversity and beta diversity analyses were performed with Qiime (v.1.9.1). Taxonomy that has significant abundance among different groups was identified by linear discriminant analysis effect size using default settings. To compare microbiome diversity among donor and recipient mice of the jejunal content transplant experiment, NMDS analysis based on Bray-Curtis dissimilarities was performed using Microbiome Analyst (v.2.0). Amplicon sequence variants were used to plot for NMDS analysis in Extended Data Fig. 3j.

### Statistical analysis

Data collection and analysis were not performed blind to the conditions of the experiments. Data distribution was assumed to be normal, but this was not formally tested. Tukey’s HSD test and two-sided or one-sided Student’s *t*-test were used to calculate *P* values, with *P* < 0.05 considered significant. False discovery rate correction was performed for metabolomics with the Benjamini and Hochberg method. Outliers were defined as values more than 1.5 times the interquartile range below quartile 1 or above quartile 3 (ref. ^100^).

### Reporting summary

Further information on research design is available in the Nature Portfolio Reporting Summary linked to this article.

## Supplementary information

Reporting Summary

Supplementary Table 1 Composition of control and inulin-supplemented diets. Inulin accounts for 10% w/w. Note that the inulin diet contains ~7% less kcal g^−1^ owing to the replacement of a small portion of corn starch with inulin.

Supplementary Table 2 LC–MS analysis results of metabolites labelled from ^13^C-fructose in liver from CF and IF. *P* values by Student’s *t*-test followed by false discovery rate correction.

Supplementary Table 3 List of enriched genes in IF and CIF relative to CF (fold change > 2, *P* < 0.05) and RNA-seq results. Genes mentioned in the Article are indicated in bold. *P* value by Student’s *t*-test followed by false discovery rate correction.

## Source data

Source Data Fig. 1 Source Data of Fig. 1

Source Data Fig. 2 Source Data of Fig. 2

Source Data Fig. 3 Source Data of Fig. 3

Source Data Fig. 4 Source Data of Fig. 4

Source Data Fig. 5 Source Data of Fig. 5

Source Data Fig. 6 Source Data of Fig. 6

Source Data Extended Data Fig. 1 Source Data of Extended Data Fig. 1

Source Data Extended Data Fig. 2 Source Data of Extended Data Fig. 2

Source Data Extended Data Fig. 3 Source Data of Extended Data Fig. 3

Source Data Extended Data Fig. 4 Source Data of Extended Data Fig. 4

Source Data Extended Data Fig. 5 Source Data of Extended Data Fig. 5

## Data Availability

RNA-seq data are available in the Gene Expression Omnibus under accession number GSE268945. The numerical source data for all graphs and charts are included with the paper. Source data are provided with this paper.

## Code availability

No custom code was used.
