# Supplementary material for: Dietary fibre-adapted gut microbiome clears dietary fructose and reverses hepatic steatosis
Source: Nat Metab. 2025 Sep 15;7(9):1801–18. doi: 10.1038/s42255-025-01356-0 (PMC12460171; doi:10.1038/s42255-025-01356-0)
Supplement: Supplementary file 1 — Reporting Summary [file 42255_2025_1356_MOESM1_ESM.pdf]

nature portfolio

Corresponding author(s): Cholsoon Jang

Last updated by author(s): Jun 5, 2025

# Reporting Summary

Nature Portfolio wishes to improve the reproducibility of the work that we publish. This form provides structure for consistency and transparency in reporting. For further information on Nature Portfolio policies, see our Editorial Policies and the Editorial Policy Checklist.

## Statistics

For all statistical analyses, confirm that the following items are present in the figure legend, table legend, main text, or Methods section.

| n/a | Confirmed | |
|---|---|---|
| ☐ | ☒ | The exact sample size (*n*) for each experimental group/condition, given as a discrete number and unit of measurement |
| ☐ | ☒ | A statement on whether measurements were taken from distinct samples or whether the same sample was measured repeatedly |
| ☐ | ☒ | The statistical test(s) used AND whether they are one- or two-sided<br>*Only common tests should be described solely by name; describe more complex techniques in the Methods section.* |
| ☒ | ☐ | A description of all covariates tested |
| ☒ | ☐ | A description of any assumptions or corrections, such as tests of normality and adjustment for multiple comparisons |
| ☐ | ☒ | A full description of the statistical parameters including central tendency (e.g. means) or other basic estimates (e.g. regression coefficient) AND variation (e.g. standard deviation) or associated estimates of uncertainty (e.g. confidence intervals) |
| ☐ | ☒ | For null hypothesis testing, the test statistic (e.g. *F*, *t*, *r*) with confidence intervals, effect sizes, degrees of freedom and *P* value noted<br>*Give P values as exact values whenever suitable.* |
| ☒ | ☐ | For Bayesian analysis, information on the choice of priors and Markov chain Monte Carlo settings |
| ☒ | ☐ | For hierarchical and complex designs, identification of the appropriate level for tests and full reporting of outcomes |
| ☐ | ☒ | Estimates of effect sizes (e.g. Cohen's *d*, Pearson's *r*), indicating how they were calculated |

*Our web collection on statistics for biologists contains articles on many of the points above.*

## Software and code

Policy information about availability of computer code

| | |
|---|---|
| Data collection | The following software were used for data collection:<br>Zen Blue software (Carl Zeiss)<br>Zen Black software (Carl Zeiss)<br>Thermo Cloud PCR (Thermo)<br>edgeR (Bioconductor)<br>Bowtie (BIOPAC Systems Inc)<br>Genowiz™ (Ocimum Biosolutions)<br>MAVEN (http://genomics-pubs.princeton.edu/mzroll/index.php) |

Data analysis

The following software were used for data analysis:
Zen Blue software (Carl Zeiss)
Zen Black software (Carl Zeiss)
ImageJ (Fiji version) Software (NIH)
GraphPad Prism (GraphPad Software)
MeV tm4 (TIGR)
Microbiome Analyst (McGill)
QuPath (GNU)
MAVEN (http://genomics-pubs.princeton.edu/mzroll/index.php)
Natural isotope correction was performed using the published code (PMID: 28471646)
Compound Discoverer (Thermo)

For manuscripts utilizing custom algorithms or software that are central to the research but not yet described in published literature, software must be made available to editors and reviewers. We strongly encourage code deposition in a community repository (e.g. GitHub). See the Nature Portfolio guidelines for submitting code & software for further information.

## Data

Policy information about availability of data

All manuscripts must include a data availability statement. This statement should provide the following information, where applicable:

- Accession codes, unique identifiers, or web links for publicly available datasets
- A description of any restrictions on data availability
- For clinical datasets or third party data, please ensure that the statement adheres to our policy

The RNA-seq data are available in the NCBI Gene Expression Omnibus under the accession number GSE268945. All other data that support the findings of this study are available from the corresponding author upon reasonable request.

## Research involving human participants, their data, or biological material

Policy information about studies with human participants or human data. See also policy information about sex, gender (identity/presentation), and sexual orientation and race, ethnicity and racism.

| | |
|---|---|
| Reporting on sex and gender | This study does not include human research participants. |
| Reporting on race, ethnicity, or other socially relevant groupings | This study does not include human research participants. |
| Population characteristics | This study does not include human research participants. |
| Recruitment | This study does not include human research participants. |
| Ethics oversight | This study does not include human research participants. |

Note that full information on the approval of the study protocol must also be provided in the manuscript.

# Field-specific reporting

Please select the one below that is the best fit for your research. If you are not sure, read the appropriate sections before making your selection.

☒ Life sciences ☐ Behavioural & social sciences ☐ Ecological, evolutionary & environmental sciences

For a reference copy of the document with all sections, see nature.com/documents/nr-reporting-summary-flat.pdf

# Life sciences study design

All studies must disclose on these points even when the disclosure is negative.

| | |
|---|---|
| Sample size | For our in vivo experiments, 6-9 mice were sacrificed per experiment group. Specific sample size for each experiment is indicated in the manuscript. This sample size was determined based on the minimum number of animals being sacrificed while at the same time, fulfilling statistically. |
| Data exclusions | For our experiment, we used ethically minimum number of mice that are statistically significant. Each group of mice were housed in controlled environment and they were of same gender and age. Therefore, we did not exclude any mouse from any group in the experiments that were carried out unless they were succumbed. Data point outliers were determined by 1.5 times the interquartile range (1.5*IQR below Q1 or 1.5*IQR above Q3). |
| Replication | All experiments conducted in this study were reproducible through repeated experiments. Unless otherwise indicated, experiments were replicated independently at least twice. |

| | |
|---|---|
| Randomization | All mice were allocated in random. No bias in sample allocation was involved. |
| Blinding | The investigators were not blinded during data collection. |

# Reporting for specific materials, systems and methods

We require information from authors about some types of materials, experimental systems and methods used in many studies. Here, indicate whether each material, system or method listed is relevant to your study. If you are not sure if a list item applies to your research, read the appropriate section before selecting a response.

## Materials & experimental systems

| n/a | Involved in the study | |
|---|---|---|
| ☐ | ☒ | Antibodies |
| ☒ | ☐ | Eukaryotic cell lines |
| ☒ | ☐ | Palaeontology and archaeology |
| ☐ | ☒ | Animals and other organisms |
| ☒ | ☐ | Clinical data |
| ☒ | ☐ | Dual use research of concern |
| ☒ | ☐ | Plants |

## Methods

| n/a | Involved in the study | |
|---|---|---|
| ☒ | ☐ | ChIP-seq |
| ☒ | ☐ | Flow cytometry |
| ☒ | ☐ | MRI-based neuroimaging |

## Antibodies

| | |
|---|---|
| Antibodies used | Anti-4-hydroxynonenal (4-HNE) antibody (clone 12F7; Invitrogen, 1:200), Anti-mouse secondary antibody conjugated to Alexa-Fluor 488 (1:1000; Jackson ImmunoResearch). |
| Validation | All the antibodies were validated for the species and applications (immunohistochemistry) by the correspondent manufacturer, which is described in the manufacturer's website. Our usage was described in the Methods section of the manuscript accordingly. |

## Animals and other research organisms

Policy information about studies involving animals; ARRIVE guidelines recommended for reporting animal research, and Sex and Gender in Research

| | |
|---|---|
| Laboratory animals | Animal studies followed protocols approved by the Institutional Animal Care and Use Committee of the University of California, Irvine. 8–week-old male C57BL/6 mice were purchased from Jackson Laboratory. Khk-C transgenic mice were bred with Villin-Cre mice (stock no. 004586; Jackson Laboratory) to generate intestine-specific Khk-C transgenic mice. Mice were group-housed on a normal light-dark cycle (7:00–19:00) with free access to chow and water. |
| Wild animals | No wild animals were captured for our study. Our mice samples were provided by approved mouse vendor. |
| Reporting on sex | We only used male mice for our study because MASLD is more prevalent in men than in women. |
| Field-collected samples | No wild animals were field-collected in our study. |
| Ethics oversight | All mouse experiments were approved and performed under institutional guidelines of the Institutional Animal Care and Use Committee of the University of California, Irvine. |

Note that full information on the approval of the study protocol must also be provided in the manuscript.

## Plants

| | |
|---|---|
| Seed stocks | N/A |
| Novel plant genotypes | N/A |
| Authentication | N/A |
